# Learning metabolic dynamics from irregular observations by Bidirectional Time-Series State Transfer Network

**DOI:** 10.1128/msystems.00697-24

**Published:** 2024-07-26

**Authors:** Shaohua Xu, Ting Xu, Yuping Yang, Xin Chen

**Affiliations:** 1 School of Basic Medical Sciences and the First Affiliated Hospital Department of Radiation Oncology, Zhejiang University School of Medicine, Hangzhou, China; 2 Zhejiang Provincial Key Laboratory for Microbial Biochemistry and Metabolic Engineering, Hangzhou, China; CNRS Delegation Bretagne et Pays de Loire, Nantes, France

**Keywords:** metabolic dynamics, neural network, time-series modeling, irregular observation, bidirectional time-series state transfer network

## Abstract

Modeling microbial metabolic dynamics is important for the rational optimization of both biosynthetic systems and industrial processes to facilitate green and efficient biomanufacturing. Classical approaches utilize explicit equation systems to represent metabolic networks, enabling the quantification of pathway fluxes to identify metabolic bottlenecks. However, these white-box models, despite their diverse applications, have limitations in simulating metabolic dynamics and are intrinsically inaccurate for industrial strains that lack information on network structures and kinetic parameters. On the other hand, black-box models do not rely on prior mechanistic knowledge of strains but are built upon observed time-series trajectories of biosynthetic systems in action. In practice, these observations are typically irregular, with discontinuously observed time points across multiple independent batches, each time point potentially containing missing measurements. Learning from such irregular data remains challenging for existing approaches. To address this issue, we present the Bidirectional Time-Series State Transfer Network (BTSTN) for modeling metabolic dynamics directly from irregular observations. Using evaluation data sets derived from both ideal dynamic systems and a real-world fermentation process, we demonstrate that BTSTN accurately reconstructs dynamic behaviors and predicts future trajectories. This approach exhibits enhanced robustness against missing measurements and noise, as compared to the state-of-the-art methods.

**IMPORTANCE:**

Industrial biosynthetic systems often involve strains with unclear genetic backgrounds, posing challenges in modeling their distinct metabolic dynamics. In such scenarios, white-box models, which commonly rely on inferred networks, are thereby of limited applicability and accuracy. In contrast, black-box models, such as statistical models and neural networks, are directly fitted or learned from observed time-series trajectories of biosynthetic systems in action. These methods typically assume regular observations without missing time points or measurements. If the observations are irregular, a pre-processing step becomes necessary to obtain a fully filled data set for subsequent model training, which, at the same time, inevitably introduces errors into the resulting models. BTSTN is a novel approach that natively learns from irregular observations. This distinctive feature makes it a unique addition to the current arsenal of technologies modeling metabolic dynamics.

## INTRODUCTION

Biosynthetic systems comprise a diverse range of substances, such as nucleic acids, proteins, enzymes, and metabolites. These substances undergo chemical reactions following kinetic principles through interaction networks to drive cellular metabolism and functioning (1, 2). Modeling metabolic systems to simulate and predict the dynamic behaviors of biosynthetic systems can help us decipher complex interactions, redesign biosynthetic pathways, and optimize production efficiency (3, 4).

Currently, two categories of methods are widely used in modeling metabolic dynamics. The first one utilizes systems of equations to describe the synthesis and degradation of metabolites within a biosynthetic system (5, 6). We refer to them as the white-box models. These models are characterized by well-defined metabolic network structures and accurate kinetic parameters, typically formulated with collections of ordinary differential equations (ODEs) or partial differential equations. Such models enable the prediction of future trajectories of metabolites constrained on given initial conditions (7). However, due to the limited availability of kinetic data as model parameters, these models typically characterize only a small number of metabolites and are unable to simulate the effect of unknown substances in complex systems (8). Recent studies have proposed various strategies for model parameter estimation (9), including statistical methods (10–14) and machine learning-assisted methods (15–21). While these approaches work well in well-studied model strains, like *Escherichia coli* (9, 13, 14, 17, 20, 21) and yeast (12, 18), they have limited applicability for non-model strains, like the antibiotic-producing *Streptomyces*, due to the lack of structural information of biological systems, i.e., comprehensive list of metabolic reactions with stoichiometric parameters and structures of metabolic pathways. In this context, genome-scale metabolic models (GSMMs) have been developed as an important supplement (22, 23). By integrating a range of constraints and perturbations, GSMMs can quantify pathway fluxes, thereby identifying bottlenecks in metabolic networks and forecasting biomass growth at steady state (24). Nonetheless, GSMMs are essentially inferred models, wherein the intrinsic inaccuracies associated with structural inference limit their accuracy in prediction. Furthermore, flux analyses are unable to simulate the dynamic behaviors of a metabolic system and are therefore not helpful in system dynamics-based decision making, such as determining the optimal harvesting time or adaptively adjusting process parameters in response to observed system states. These limitations require an alternative modeling strategy to address (25).

Another category of methods involves the usage of statistical modeling or machine learning methods to learn from observed time-series trajectories (26). They directly learn the dynamic behaviors of a system from observations, using inexplainable latent variables to simulate the system mechanisms. We refer to them as the black-box models, which include regression, Bayesian network, and neural network (27–29). Unlike white-box models, black-box models are constructed from temporal observations of a biosynthetic system, without relying on prior knowledge of its network structure and kinetic parameters of the reactions. Recent advances in measurement techniques have enabled convenient system-level characterization of RNA dynamics, e.g., SLAM Seq (30), TimeLapse-seq (31) and TUC-seq (32), and metabolite dynamics, e.g., RTMet (33) and CIVM-NMR (34). These technologies notably expanded the type and amount of acquirable time-series data, which also demand parallel advances in analysis methods to learn from them.

Among existing approaches that have been developed for learning from multi-dimensional time-series trajectories, deep neural networks demonstrated remarkable proficiency and are considered state-of-the-art. For instance, the recurrent neural network (RNN, a widely used framework for dealing with time series, has been successfully applied in a range of tasks including imputation (35, 36), prediction (37, 38), and classification (39). Another noteworthy model is the Transformer, which is based on multi-head self-attention mechanisms and is capable of processing each element in sequence in parallel while paying attention to global contextual information (40). It has been proven highly efficient in the areas of natural language processing (41), computer vision (42, 43), and time-series tasks (44–46).

For most black-box models, training data are expected to be complete and accurate, which we referred to as regular. However, observed time-series trajectories of a fermentation process are typically irregular, consisting of multiple independent batches, each batch consisting of discontinuously observed time points, and each time point potentially containing missing measurements. Some of these irregularities stem from the measurement technologies and are therefore an intrinsic feature of observed data. If training data are irregular, pre-processing steps are typically required to fill in the missing time points and measurements. In this process, however, imputation errors will be simultaneously introduced into training data, thereby limiting the accuracy of the resulting model.

To address this issue, we present the Bidirectional Time-Series State Transfer Network (BTSTN), which is designed to model system dynamics directly from irregularly observed time-series trajectories. Its structure comprises one featurizer unit to represent the system state (in the feature space) at each time point, irrespective of whether it is observed, and two generator units to represent the transfer functions that compute the system state at a given time point from its previous or next time point. These units are then serially connected to represent the temporal relationship between any two time points. With this design, only the reliably observed measurements are used to calculate the loss function and initiate back-propagation. As a result, BTSTN natively learns from irregular observations and therefore is free from the training noise introduced in the pre-processing imputation step. This approach has applicability in scenarios where observations are irregular and available pre-processing methods are not sufficiently accurate.

## RESULTS

### Dynamics reconstruction with irregular observations

As detailed in the Materials and Methods, BTSTN is a novel neural network design without prior application studies. Therefore, we first evaluated the feasibility of such design with a simulated ideal dynamic system. Relying on theoretical data sets allows us to control unexpected confounding factors, and may help us illustrate the effectiveness, accuracy, and robustness of BTSTN with well-characterized parameters.

**Fig 1:**
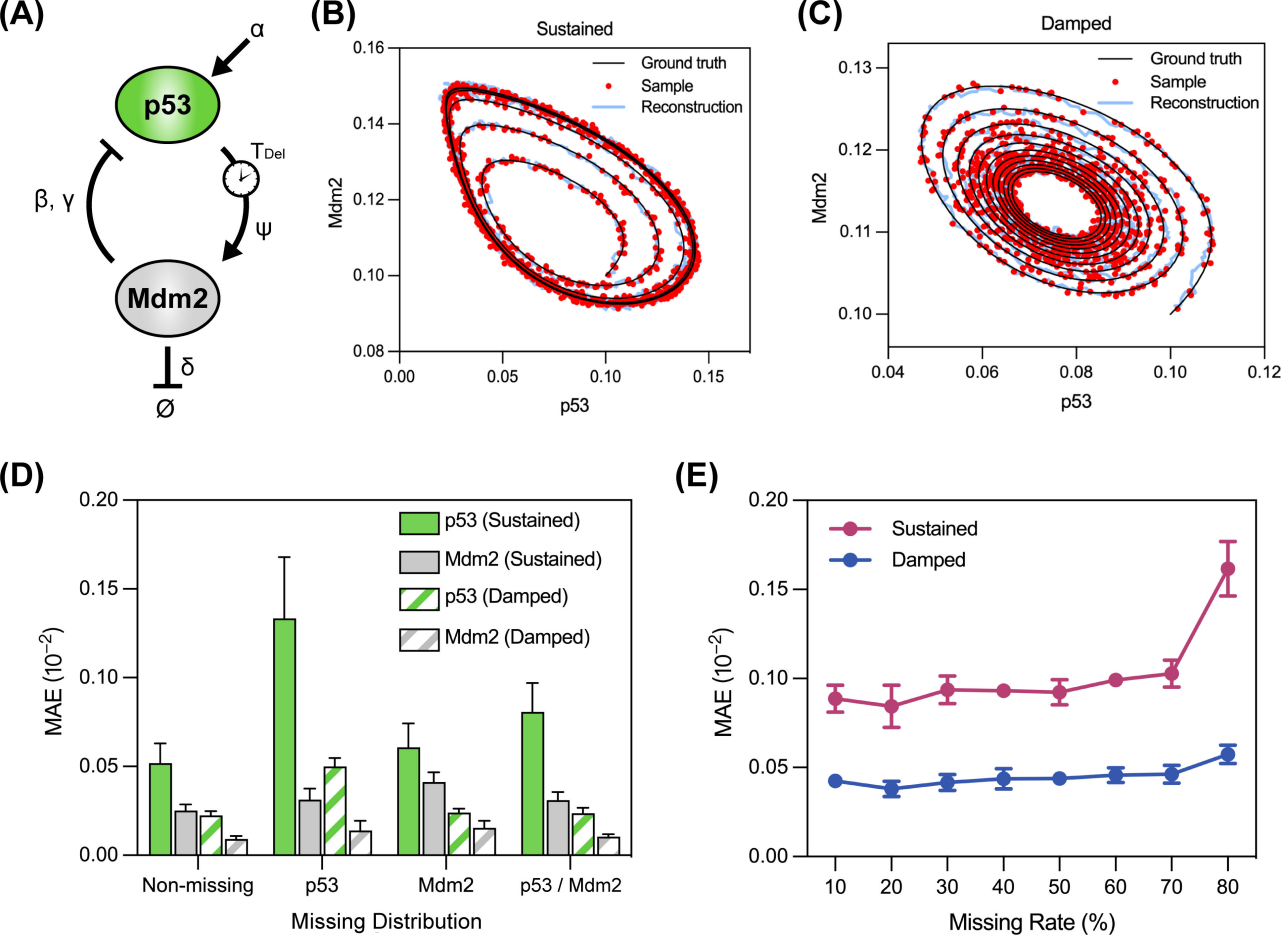
Reconstruction of the p53-Mdm2 dynamic system with incomplete and noisy data. (**A**) Schematic diagram of the p53-Mdm2 oscillator featuring a negative feedback loop, represented with the parameters (α, β, γ, ψ, δ, and *T*_Del_) in the mathematical model. (**B and C**) System reconstruction with BTSTN for the SUSTAINED and DAMPED data sets, respectively. The training data (red dots) are masked with a 30% missing rate and added with Gaussian noise (standard deviation 0.01) from the ground truth (black curves). The light blue curves are the reconstructed results of all time points. (**D**) Evaluation of the BTSTN performance on the SUSTAINED and DAMPED data sets with different missing patterns. (**E**) Evaluation of the BTSTN performance on the SUSTAINED and DAMPED data sets with Gaussian noise (standard deviation 0.01) across different missing rates from 10% to 80%. MAE is illustrated as the error performance metric.

Dynamics reconstruction, which involves restoring the dynamic behaviors of a system, is a common task used to evaluate time-series modeling approaches (47). Here, we evaluated the reconstruction capability of BTSTN with partially observed and noisy time-series trajectories. For this purpose, we used a well-known biological system for generating simulated system dynamics, the p53-Mdm2 dynamic system (48, 49). This system features a negative feedback regulation with an explicit delay parameter (*T*_Del_) which determines the oscillation pattern of the system (Fig. 1A). Two time-series trajectories showing the sustained and damped oscillation pattern were generated with two delay parameters, each trajectory including 2,000 time points. These two cases were called the SUSTAINED and DAMPED data sets. Next, we randomly masked 30% of the measurements from these trajectories and added a Gaussian noise with 0.01 standard deviation to all measurements. Then, BTSTN was used to learn from these irregular data sets. After models were trained, all featurizer (*F*) units were decoded with the decoder (*D*) unit to obtain reconstructed trajectories. Three metrics (i.e., mean absolute error [MAE], root mean square error [RMSE], and mean relative error [MRE]) were used to compare the reconstructed values with the ground truth. Results showed that BTSTN has successfully reconstructed the dynamic behaviors of both sustained and damped oscillations (Fig. 1B and C; Fig. S2).

After observing that BTSTN was able to reconstruct oscillation patterns in the presence of missing measurements, we set out to investigate whether the distribution of missing measurements (i.e., missing pattern) affects its performance. For this purpose, we randomly masked either 60% of p53 measurements or 60% of Mdm2 measurements from the SUSTAINED and DAMPED data sets, which resulted in the SUSTAINED-P53, SUSTAINED-MDM2, DAMPED-P53, and DAMPED-MDM2 data sets. In these data sets, missing happened with a single variable. Then, we randomly masked 30% of all measurements from the SUSTAINED and DAMPED data sets, which resulted in the SUSTAINED-ALL and DAMPED-ALL data sets. In these data sets, missing happened with all variables, with the same total number of missing measurements in the previous data sets. BTSTN was used to reconstruct system dynamics from all these data sets. Results showed that missing data from a single variable lead to greater reconstruction error for that particular variable, and BTSTN exhibited better accuracy in the damped system compared to the sustained system. In addition, the p53 measurements were more informative in reducing the overall reconstruction error compared to the Mdm2 measurements. These observations suggest that the distribution of missing measurements may indeed affect the performance of BTSTN (Fig. 1D; Table S1). In addition, we evaluated the reconstruction accuracy of BTSTN across different levels of missing rate. Notably, even when the missing rate reached a high level of 80%, BTSTN still reconstructed system dynamics with satisfactory accuracy (Fig. 1E; Table S2).

### Prediction of future trajectories

In addition to dynamics reconstruction, predicting future trajectories is another major challenge for modeling system dynamics. To evaluate the accuracy of BTSTN in predicting future trajectories, we utilized a more complex ideal system for generating simulated system dynamics, the $\sigma^{28}$-TetR mRNA/protein regulatory system (50). Ten time-series trajectories were simulated with different initial conditions, eight of which (Batches 1–8) were used for model training and two of which (Tests 1 and 2) were used for tests (Fig. S3).

**Fig 2:**
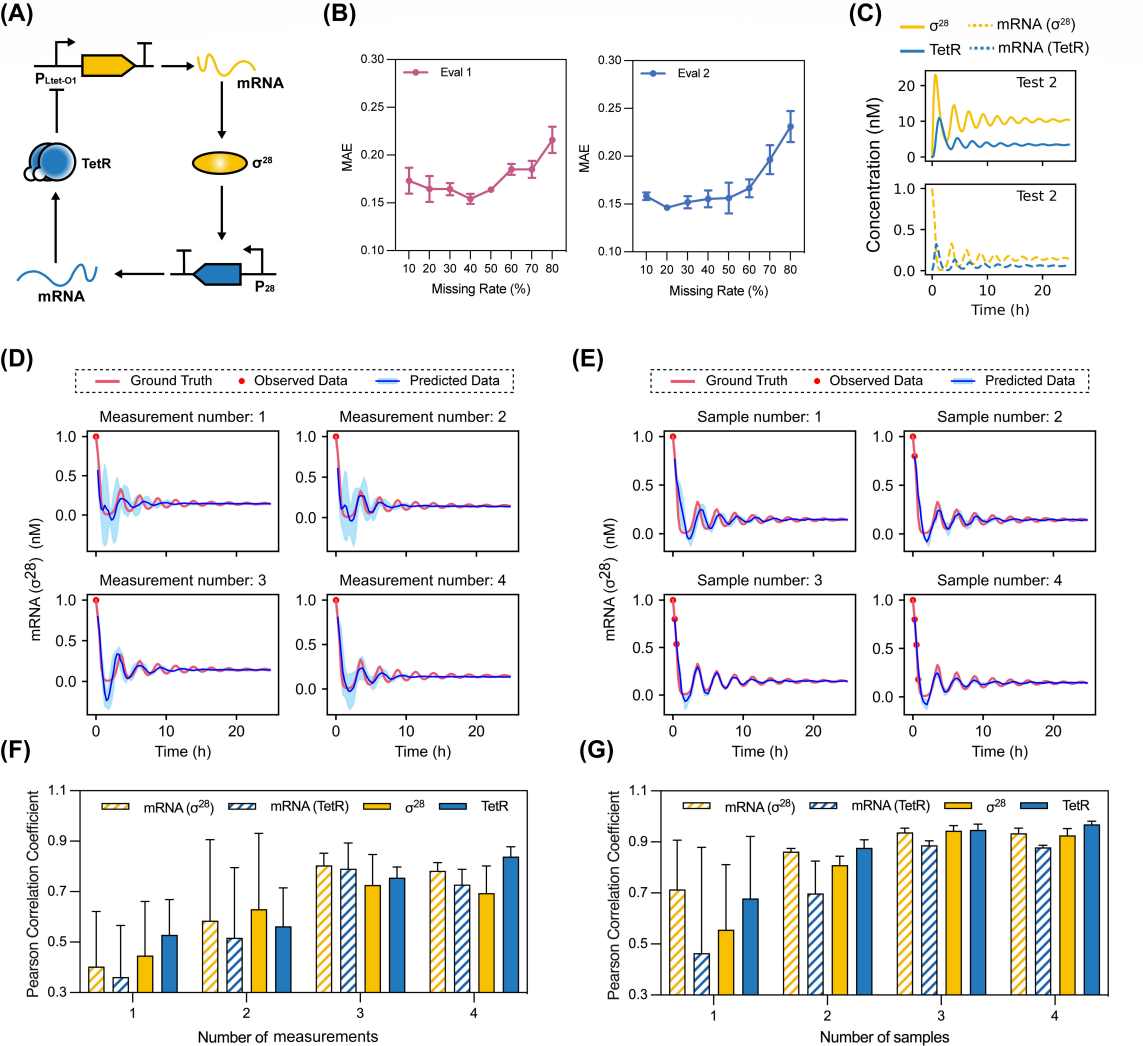
Prediction of the unknown time-series trajectories in the $\sigma^{28}$-TetR dynamic system. (**A**) Schematic diagram of the $\sigma^{28}$-TetR mRNA/protein regulatory oscillator featuring a negative feedback loop where $\sigma^{28}$ and TetR act as activator and repressor, respectively. (**B**) Extrapolation performance [first evaluation (Eval 1) and second evaluation (Eval 2)] of BTSTN on the unknown time-series trajectories (Tests 1 and 2) across different missing rates from 10% to 80%. MAE is used as the error performance metric. (**C**) Simulated trajectories of mRNA and proteins of $\sigma^{28}$ and TetR in the Test 2 data set. (**D and E**) Predicted mRNA trajectories of $\sigma^{28}$ (blue curves) based on the first time point of the Test 2 data set: provided with one to four measurements (red dots in panel D) and with one to four continuous time points (red dots in panel E) from the ground truth (red curves), respectively. The shaded region (light blue) corresponds to ±1 standard deviation. (**F and G**) Pearson correlation between the predicted and actual trajectories in the Test 2 data set: provided with one to four measurements (**F**) and with one to four continuous time points (**G**), respectively.

First, we assessed the predictive accuracy of BTSTN on unknown (non-training) trajectories. In the first evaluation, eight models trained on masked training data sets (Batches 1–8) with eight levels of missing rates (i.e., masked 10%–80% of the data) were evaluated for their accuracy in predicting 50% of the randomly masked data in test data sets (Tests 1 and 2). In the second evaluation, a model trained with the entire training data sets (i.e., the FULLY-TRAINED model) was evaluated for its accuracy in predicting different levels of masked data (10%–80%) in test data sets. In both experiments, BTSTN demonstrated accurate and consistent prediction (Fig. 2B; Table S3).

Then, we used the FULLY TRAINED model to predict the future trajectories of the Test 2 data set based on its first four time points. Results showed that the prediction accuracy increased, and the corresponding confidence interval narrowed with the number of available time points (Fig. 2E and G) and measurements (Fig. 2D and F) increased. It was noteworthy that the prediction accuracy stabilized after three time points were provided. In addition, we investigated whether discontinuity of provided time points may have an impact on prediction accuracy. For this purpose, we used the FULLY TRAINED model to predict future trajectories of the Test 2 data set based on four discontinuous time points, i.e., the first, second, fourth, and seventh time points. Similarly, the results demonstrated an increase in both prediction accuracy and stability when more time points were provided (Fig. S4). The accuracies were similar to those when continuous time points (i.e., first, second, third, and fourth time points) were provided (Fig. 2E). Furthermore, when we randomly masked one to two measurements from each known time point, BTSTN also exhibited a comparable pattern with only a slightly lower accuracy (Fig. S5).

### Robustness against missing measurements and noise

As mentioned in the Introduction, observations of the time-series trajectory of a biosynthetic system are intrinsically irregular. Therefore, robustness against missing measurements and noise is an essential feature for solutions relying on such data. For this purpose, we compared BTSTN with several traditional and state-of-the-art time-series modeling solutions, including four statistical methods, i.e., mean, *k*-nearest neighbor (KNN), matrix factorization (MF) (51), and multiple imputation by chain equations (MICE) (52), and three deep learning models, i.e., bidirectional recurrent imputation for time series (BRITS) (53), Transformer (40), and self-attention-based imputation for time series (SAITS) (47). The ideal dynamic system $\sigma^{28}$-TetR mRNA/protein regulatory system that was used previously was also used for subsequent evaluation.

**Fig 3:**
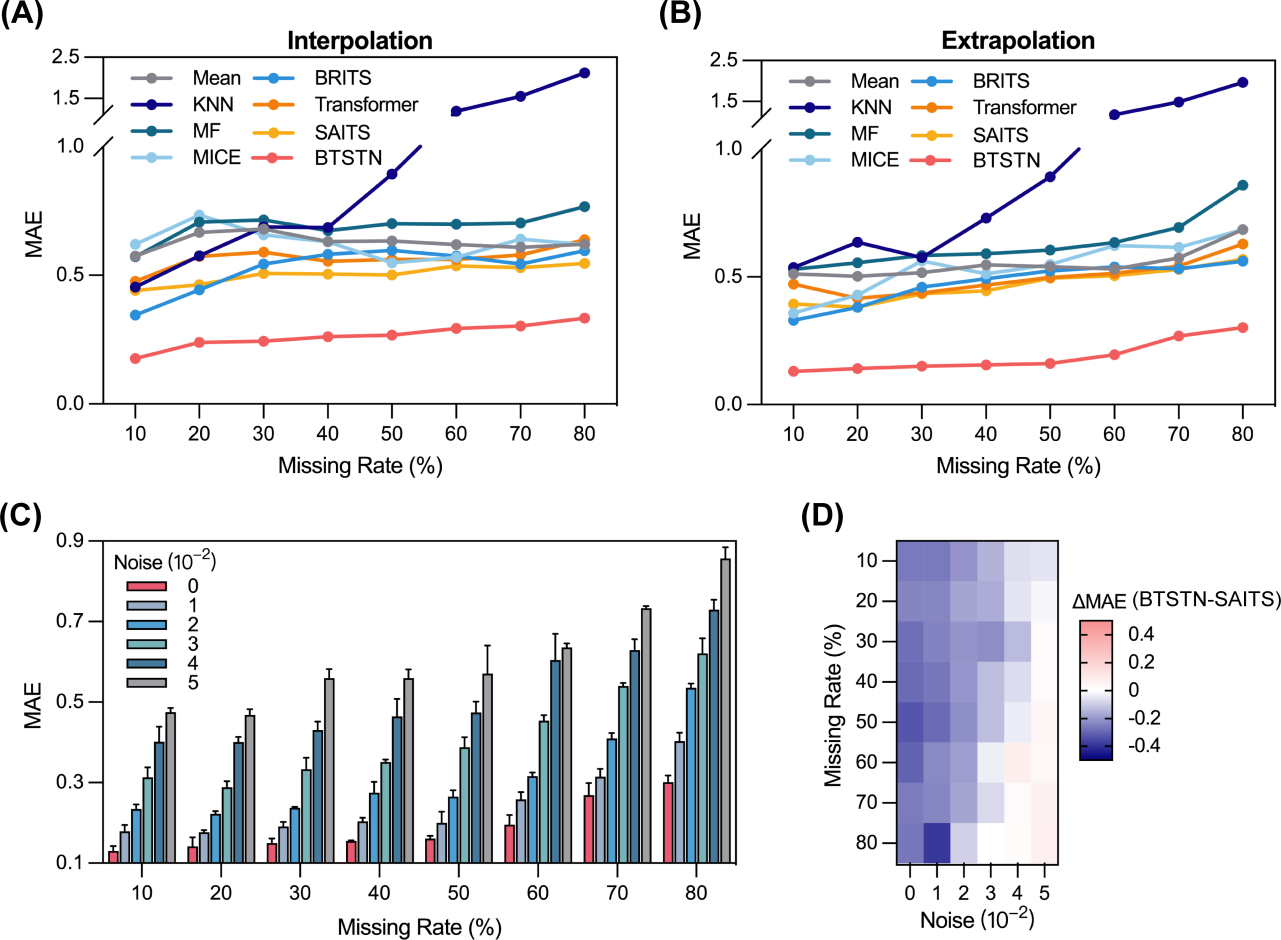
Robustness of BTSTN against missing measurements and noise. (**A and B**) Comparison of interpolation and extrapolation performance between BTSTN and seven baseline methods, including four naive methods (mean, KNN, MF, and MICE) and three state-of-the-art (SOTA) deep learning models (BRITS, Transformer, and SAITS). (**C**) Evaluation of BTSTN performance on the data sets with Gaussian noise of different levels (standard deviation from 0.01 to 0.05). (**D**) Comparison between BTSTN and SAITS model. The color scale indicates the MAE differences. Blue and red indicate that BTSTN and SAITS, respectively, have higher accuracy respectively. All analyses are evaluated across different missing rates from 10% to 80%. MAE is used as the error performance metric.

First, we randomly masked 10%–80% of the measurements in both training data sets (Batches 1–8) and test data sets (Tests 1 and 2) to simulate the missingness. We compared the interpolation and extrapolation accuracies of different methods across different missing rates (10%–80%). The statistical methods were directly applied to predict the masked data in both training and test data sets. The deep learning models trained on masked training data sets were used to interpolate the missing data, while the models trained on complete training data sets were used to extrapolate the missing data in the test data sets. Results showed that deep learning methods in general outperformed statistical methods across all missing rates. BTSTN achieved the highest performance, while the second place was taken by SAITS, a Transformer-based time-series modeling approach (Fig. 3A and B; Tables S4 and S5).

Then, we added Gaussian noise at different levels (standard deviations from 0.01 to 0.05) to all masked data sets and used them for training BTCN models. The resulting models were subsequently applied to extrapolate missing data in test data sets with the same missing rates and noise levels as their respective training data sets. Results showed a general increase in extrapolation errors with higher missing rates, with insignificant increments at low missing rates ranging between 10% and 50% (Fig. 3C; Table S6). In comparison, SAITS, the approach second to BTSTN in prediction accuracy, was also evaluated for its robustness against noise. Notably, BTSTN demonstrated a substantial advantage over SAITS at most of the missing rates and noise levels. Only at extremely high missing rates and noise levels did these two approaches exhibit similar performance (Fig. 3D; Table S7).

### The effect of time interval on model accuracy

**Fig 4:**
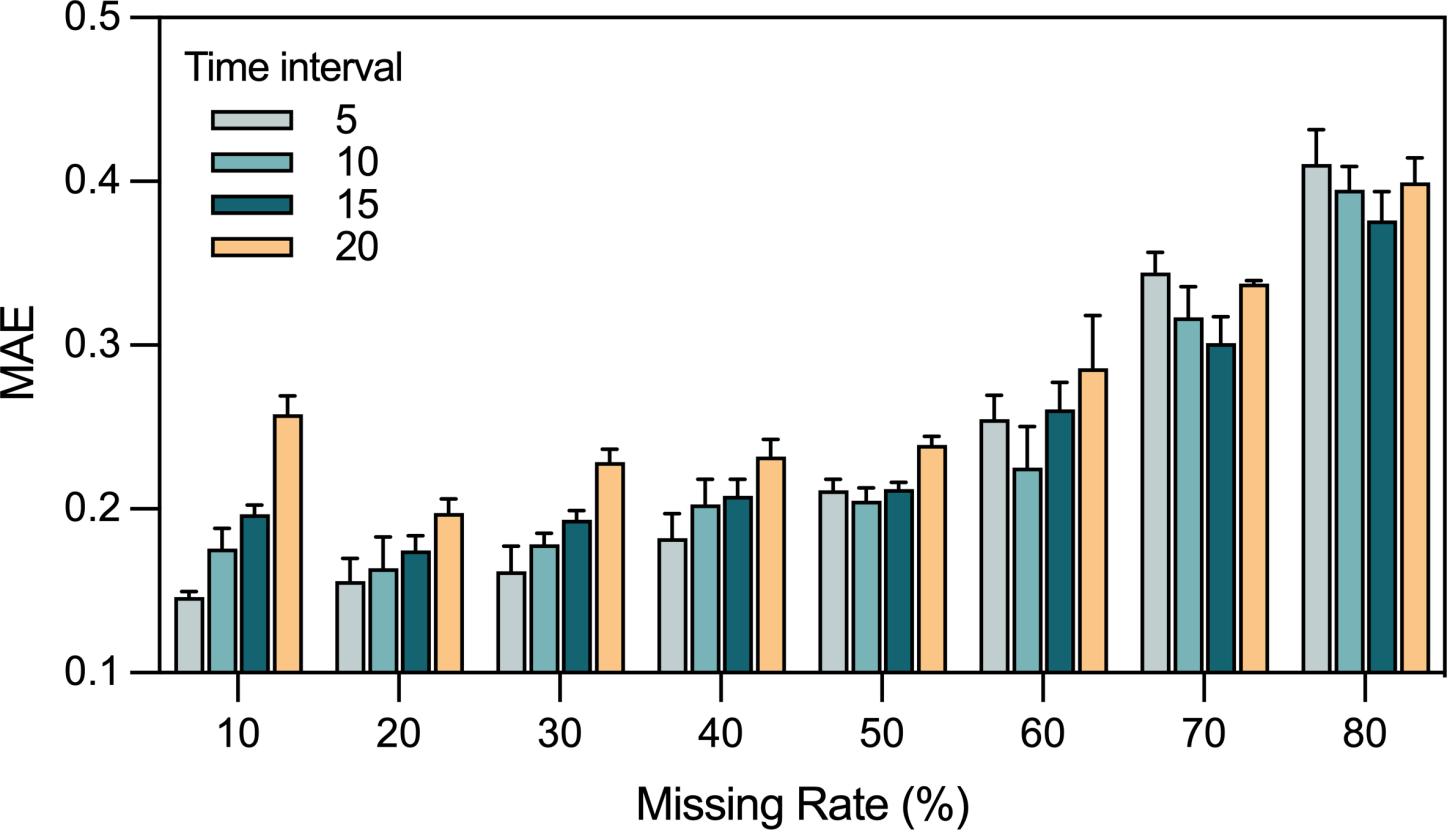
Evaluation of the impact of time intervals (5, 10, 15, and 20) on BTSTN performance. MAE is used as the error performance metric.

As detailed in the methods section, BTSTN learns from observations of the system state conversion between any two time points. Therefore, limiting the time interval (i.e., the number of time units between the starting and ending time points) may affect model accuracy. To characterize this effect, we limited the maximum intervals to four levels (i.e., 5, 10, 15, and 20) during model training. First, the training data sets with masked measurements ranging from 10% to 80% and Gaussian noise (standard deviation 0.01) were used for training. Then, the resulting models were used to extrapolate the missing data in the test data sets with the same missing rates and noise levels as their respective training data sets. Results showed that, with a maximum interval of 5, lower missing rates correlated with higher accuracy, while with higher maximum intervals, the optimal performance was consistently observed at a missing rate of 20% (Fig. 4; Table S8).

Moreover, reducing the maximum interval for training improved model accuracy for data sets with lower missing rates (10%–40%) but not for those with higher missing rates (50%–80%). For example, in training data sets with 50%–60% missing measurements, a maximum interval of 10 achieved the highest accuracy, whereas in training data sets with 70%–80% missing measurements, a maximum interval of 15 yielded optimal results. However, the optimal performance was not achieved with a maximum interval of 20 across all training data sets. This suggests that with larger learning intervals, the accumulated errors that occur during numerical computations may offset the benefits of additional information gained for training. Therefore, a reasonable time interval of system state conversion used in model training is necessary for optimal model accuracy.

### Modeling succinic acid fermentation process

**Fig 5:**
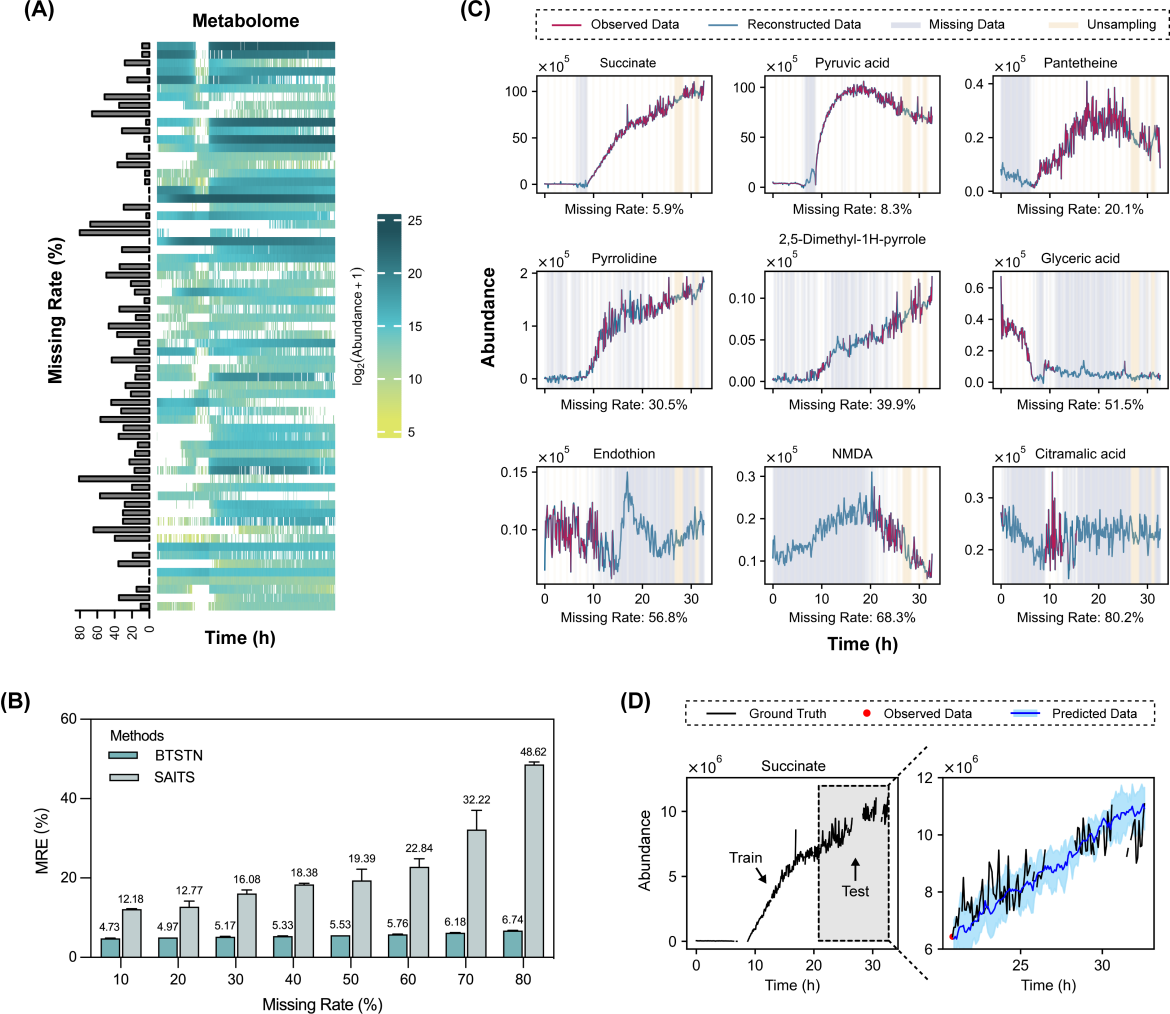
Application of BTSTN on the succinate fermentation data set. (**A**) Abundance of metabolites from online mass spectrometry. The annotated metabolites are shown by rows. The sample collection time points are shown by columns. Missing measurements are indicated. (**B**) Comparison of interpolation performance between BTSTN and SAITS models. MRE is used as the error performance metric. (**C**) Reconstruction of the trajectories of nine metabolites (dark green curves) with BTSTN based on the available observations (dark red curves). The light yellow and light blue areas indicate the unsampled time points and the missing values of corresponding metabolites, respectively. (**D**) Predicted trajectory of succinic acid (blue curve) based on the first time point (red dot) in the test data set.

With ideal dynamic systems, we have demonstrated the potential advantage of BTSTN in modeling irregular observations. In this work, we applied BTSTN to a real-world data set from a fermentation process. Cortada-Garcia et al. established an online untargeted metabolomics approach and monitored a batch of succinic acid fermentation by *Escherichia coli* (33). Over 33 hours, 338 samples were collected, with 67 metabolites confidently annotated and quantified by liquid chromatography-mass spectrometry (LC-MS) signals (Fig. 5A). As previously mentioned, measurements produced by this category of observation technology had a probability of missing readings. In this case, 25.5% of the measurements were missing.

Similarly, in this data set, we first randomly masked 10%–80% of the observed data and evaluated how well BTSTN and SAITS were able to learn and interpolate these masked data. Results showed that BTSTN maintained a stable and high interpolation accuracy across all levels of missing rates, significantly outperforming SAITS. The latter exhibited an expected trend where the accuracy increased with decreasing missing rates (Fig. 5B; Table S10). Furthermore, we used BTSTN to reconstruct the original trajectories of all 67 metabolites (Fig. 5C; Fig. S6 and S7). The reconstructed measurements aligned well with our intuitive expectation of their potential ranges.

Then, we evaluated the prediction accuracy of BTSTN with this data set. For this purpose, we separated the observed trajectory into two data sets: the first 250 time points made the training data set, and the remaining 88 time points made the test data set. A BTSTN model trained with the training data set was subsequently used to predict the trajectory of all metabolites in the test data set based on its first time point. As an example, we presented the predicted trajectory of the primary product of this fermentation, succinate (Fig. 5D). Results demonstrated a close alignment between the predicted trajectory and the observed trajectory, with the confidence interval of prediction covering the observed measurement.

### Extension to the linear dynamic systems

We have demonstrated the potential advantage of BTSTN modeling metabolic systems, which are generally considered non-linear systems. It was interesting to explore further whether BTSTN is a viable choice for modeling linear dynamic systems.

**Fig 6:**
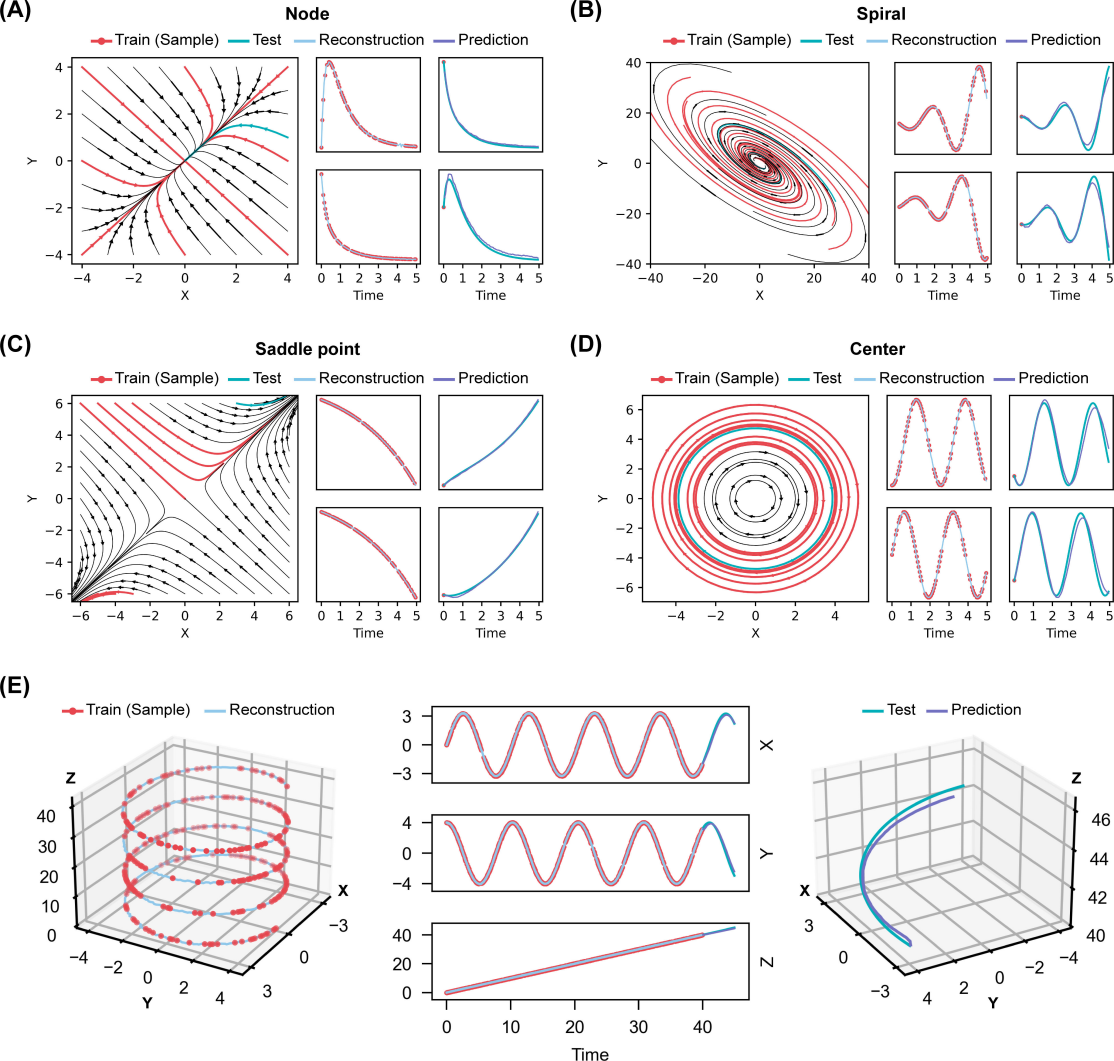
Extended applicability of BTSTN for linear systems. (**A–D**) Reconstruction and prediction of four linear systems based on different types of fixed points. In each system, multiple data sets of ground truth (black curves) can be obtained with different initial values of *X* and *Y*. Within each data set, eight trajectories (red curves) and one trajectory (green curve) are selected from ground truth as training and test data sets, respectively. Partial observations (red dots) from 70% of training data are used for BTSTN training. The trained BTSTN is used to reconstruct the system (light blue curve) and to predict (purple curve) the future part of the test trajectory based on the first time point. (**E**) An artificial three-dimensional linear system is further introduced to evaluate the applicability of BTSTN to model a more complex linear system.

For this purpose, we first generated four data sets of two-dimensional linear systems classified by different fixed points, i.e., node, focus, saddle point, and center. Each data set comprised nine time-series trajectories, each trajectory containing 100 time points, respectively. For each data set, eight trajectories with 30% randomly masked measurements were used for training, and one trajectory for test. Results showed that in all four ideal linear systems, BTSTN precisely reconstructed their trajectories with partial observations and accurately predicted the future trajectories based on their first time point (Fig. 6A through D).

Furthermore, we constructed a more complex three-dimensional linear system, with two variables creating a stable oscillation and the third variable increasing linearly (Fig. 6E). We generated a data set containing 1,000 time points and used the first 800 time points for training. Within the training data set, we introduced a missing rate of 30% and Gaussian noise with 0.01 standard deviation. The model trained with the training data set was used to predict the remaining 200 time points for test. Once more, BTSTN was able to precisely reconstruct the masked data and predict future trajectory based on its initial time points (Fig. 6E).

## DISCUSSION

Modeling microbial metabolic dynamics enables the simulation of target product synthesis, which can aid in determining an optimal harvest time and identifying the best process strategies for high overall yields, thereby enhancing the control of fermentation processes. White-box models rely on a comprehensive understanding of the related metabolic networks and kinetic parameters for accurate simulation of system dynamics (5–7, 22–24). However, such knowledge on industrial strains is typically limited (8). Although the network structure may be inferred from model strains by homology and the parameters can be estimated through various strategies (9–21), the inferred results are intrinsically noisy, which leads to poor dynamic prediction accuracy (25). To date, white-box models are seldom used in dynamics prediction in industrial settings.

On the other hand, black-box approaches do not require prior mechanistic knowledge. They model the dynamic behaviors of a system from observations with inexplainable latent variables, making them a viable alternative for industrial applications (26–29, 35–46). However, most existing black-box approaches assume that the observed trajectories are complete and accurate. Yet as limited by both technical and cost factors, such observations are seldomly regular. For instance, in a fermentation process, some time points may provide more information than others, such as those when cells transit from their primary metabolism to secondary metabolism, or from the exponential phase to the stable phase of growth. For cost considerations, usually, expensive system state measurements are selectively performed, which inevitably leads to missing time points or measurements in observations. Also as mentioned previously, widely used measurement technologies, such as the quantification of metabolites by LC-MS, have the limitation of randomly missing measurements (33). For these reasons, pre-processing steps are generally required for existing black-box models, which, at the same time, may introduce imputation noises and compromise the accuracy of resulting models.

In this work, we present BTSTN, a novel neural network design for modeling metabolic dynamics from irregular observations. The units of the BTSTN serve different purposes. First, the *F* unit has a similar functionality as the embedding layer to create a representation of system state in feature space. On the other hand, it is different from the traditional design of embedding layer that takes observed values as input. The *F* unit takes a fixed (random) value as input, which enables BTSTN to leverage only available observed data for model training. Second, the generators (forward generator [*G*] and backward generator [*G*′]) units essentially represent state transfer functions, which simulate the molecular systems of a strain that transfer the observable system states from one time point to the next or the previous. As long as the molecular mechanisms of a strain remain consistent, the trained BTSTN model can be generalized to different initial conditions.

We evaluated the performance of BTSTN modeling irregularly observed trajectories using data sets from both ideal system dynamics and a real-world succinate fermentation process. Results showed that, even with a high level of missing rate and noise, BTSTN has effectively reconstructed the dynamic behaviors and predicted future trajectories in these systems. As shown in Fig. 3, BTSTN demonstrated a significant advantage over seven widely used statistical and deep learning methods. Notably, the second best method was SAITS, which employs the same neural network structure (i.e., Transformer), which powered ChatGPT (54) and was reported as state-of-the-art in a previous study (47).

Analysis of the BTSTN structure from the perspective of information flow suggests that BTSTN effectively imposes constraints on the weights of each edge with all observed measurements across different batches. Specifically, the edge weights of a featurizer unit, $F_{t}$, corresponding to time point t, are constrained by all observed measurements from the same batch. This is because, as detailed in the Materials and Methods, $F_{t}$ is involved in the $F-G_{\left(i\right)}-D$ and $F-G_{\left(i\right)}^{`}-D$ structures, which are trained over the observed measurements from all time points in the same batch. Additionally, the G, $G^{`}$, and D units share parameters in all $F-G_{\left(i\right)}-D$ and $F-G_{\left(i\right)}^{`}-D$ structures across all batches. Therefore, their edge weights are simultaneously constrained by all observed measurements across all batches. Consequently, the edge weights of $F_{t}$, reliant on the structure of the G, $G^{`}$, and D units for training, are in turn constrained by all observed measurements across all batches.

Because of the high efficiency of the BTSTN structure imposing constraints on edge weights, this approach has the potential to obtain an accurate model from limited observed data. As shown in Fig. 5B, when dealing with the succinate fermentation data set, SAITS showed a notable increase in accuracy as the missing rate decreased from 80% to 10%. In contrast, the performance of BTSTN remained relatively stable across different missing rates, suggesting that a small number of observations may be sufficient for BTSTN to learn the system dynamics.

To date, though metabolic network models have found applications in biosynthetic process refinement, the lack of accurate kinetic data restricts their use mainly to flux analysis, which is aimed at identifying steady-state metabolic bottlenecks and providing insights for genetic modification of production strains. However, other important aspects of sustainable and economical production, such as adaptive control of the fermentation process based on real-time system state observations or determination of the optimal harvesting time considering multiple objectives (e.g., the yield of target product, the amounts of by-products, and the cost of purification), demand accurate modeling of metabolic dynamics. A preliminary step toward accurate modeling is the establishment of a method framework that adapts to the limitations of available observation data. We envision that the addition of BTSTN to the current arsenal of dynamic modeling approaches may offer a viable basis for the development of more powerful automatic fermentation monitoring and control approaches in industrial settings. In addition, even though BTSTN cannot offer explicit genetic modification suggestions for strains, they may still provide similar insights on pathways associated with better strain performance. For instance, if simulating the effect of continuously adding a specific metabolite to the medium would always lead to increased yield, this result may suggest that relevant metabolic pathways may be promising targets for genetic modification.

## MATERIALS AND METHODS

### Problem formulation

The problem of modeling metabolic dynamics is formulated as follows. A collection of N non-continuous multivariate time-series trajectories, each involving D variables (a.k.a. measurements), are observed from a biosynthetic system in action. The data set of all trajectories is denoted as $S=\left\{s_{1},s_{2},\ldots ,s_{n},\ldots s_{N}\right\}$, in which each trajectory is represented as a time-observation pair. For the *n*th trajectory, which contains $L_{n}$ observed time points, it is denoted as $s_{n}=\left(\overset{⃑}{t_{n}},\overset{⃑}{x_{n}}\right)$ in which $\overset{⃑}{t_{n}}=\left\{t_{n}^{1},t_{n}^{2},\ldots ,t_{n}^{l},\ldots ,t_{n}^{L_{n}}\right\}\in R^{L_{n}}$ denotes all observed time points, while $\vec{x_{n}}=\left\{\vec{x_{n}^{\text{1}}},\vec{x_{n}^{\text{2}}},\ldots ,\vec{x_{n}^{l}},\ldots ,\vec{x_{n}^{L_{n}}}\;\right\}\in R^{L_{n}\times D}$ denotes the corresponding observation data. The *l*th time point of $\overset{⃑}{x_{n}}$ is denoted as $\overset{⃑}{x_{n}^{l}}=\left\{x_{n}^{l\{1\}},x_{n}^{l\{2\}},\ldots ,x_{n}^{l\{d\}},\ldots ,x_{n}^{l\{D\}}\right\}\in R^{D}$, in which any value may be missing. Accordingly, $x_{n}^{l\{d\}}$ represents the *d*th variable of the *l*th time point in the *n*th trajectory. To present the original missing information, we introduce a missing mask vector, $M=\left\{m_{1},m_{2},\ldots ,m_{n},\ldots m_{N}\right\}$, as follows:


$$\begin{array}{c} m_{n}^{l\left\{d\right\}}=\left\{ \begin{array}{ll} 1,\;\; & if\;x_{n}^{l\left\{d\right\}}\;is\;observed\\ 0,\;\; & if\;x_{n}^{l\left\{d\right\}}\;is\;missing \end{array}\right. \end{array}$$

The BTSTN method can be used for three objectives. The modeling objective is to train a model with partially observed time-series trajectories, which computes the system states at a given time point from its previous one. Then, the trained model can be used to reconstruct the training trajectories, which is referred to as the reconstruction objective. In addition, the trained model can be used to predict future (non-training) trajectories based on partial observations of their initial time points, which is referred to as the prediction objective.

### Bidirectional Time-Series State Transfer Network

**Fig 7:**
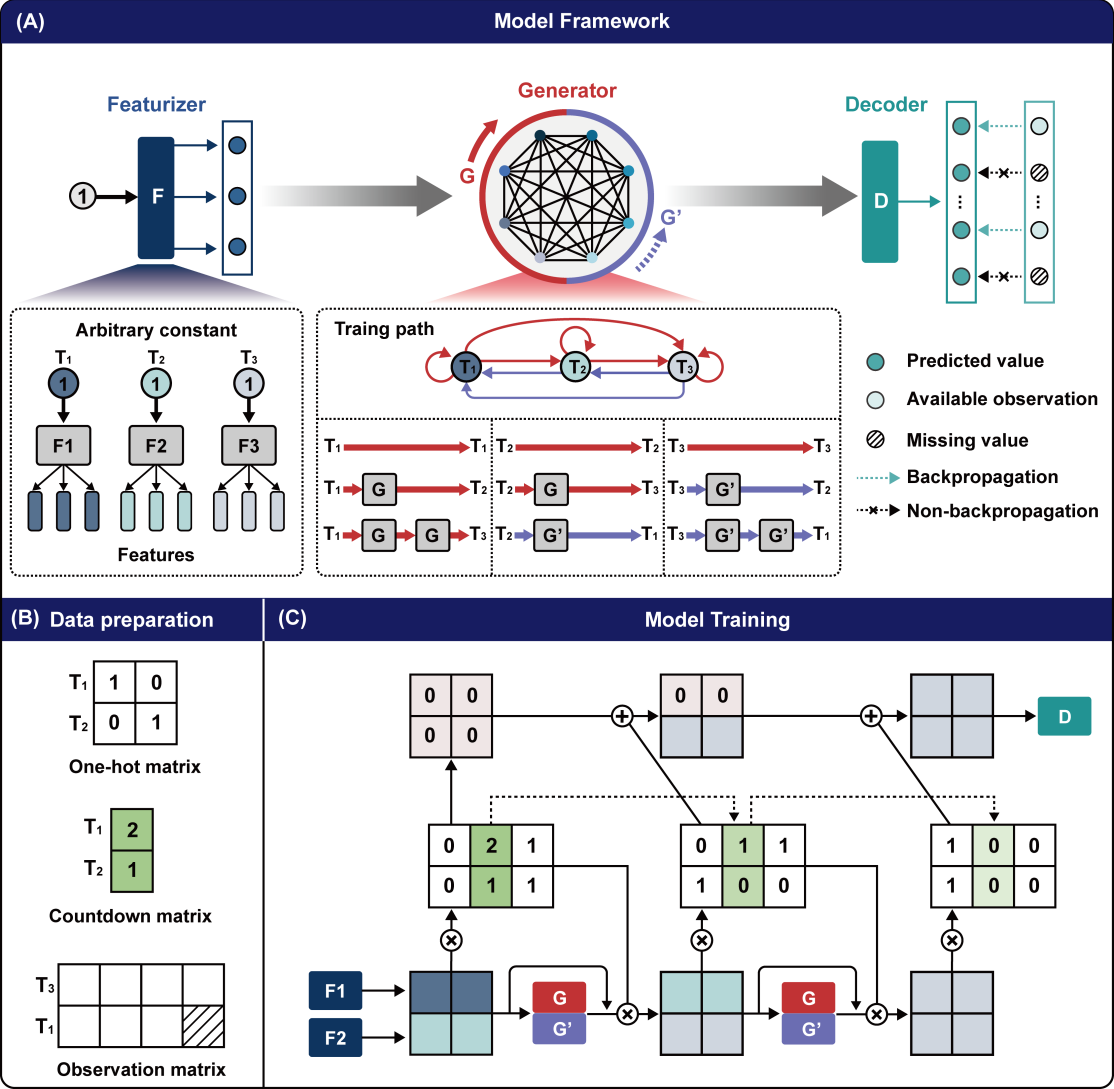
An overview of the Bidirectional Time-Series State Transfer Network (BTSTN). (**A**) A training path between any two time points in a trajectory can be established in a bidirectional manner (i.e., forward or backward) with four types of sub-units, i.e., the featurizer (*F*), the forward generator (*G*), the backward generator (*G*′), and the decoder. (**B**) Three matrices are prepared for model training including a one-hot matrix to mark the random input value of each time point, a countdown matrix to track the generation times, and an observation matrix containing randomly missing values. (**C**) During the model training, the countdown matrix is subtracted by 1 after each generation round. Upon reaching a count value of 0, the corresponding process is completed. Please refer to https://github.com/xsh93/BTSTN for implementation details.

The structure of BTSTN consists of four types of sub-units, including F, G, $G^{`}$, and *D* (Fig. 7). For each time point in each trajectory, there is a corresponding F unit to represent it. These F units are single-layer sub-networks with fixed inputs. Each F unit can be interpreted as a unique function that converts a fixed (random) input value into a K-dimensional feature vector that describes the system states (in the feature space) at each time point. We denote $E=\left\{e_{1},e_{2},\ldots ,e_{n},\ldots e_{N}\right\}$ as the feature space trajectory corresponding to the observation space trajectory S. Therefore, correspondingly, the *n*th feature space trajectory is denoted as $e_{n}=\left\{\vec{e_{n}^{\text{1}}},\vec{e_{n}^{\text{2}}},\ldots ,\vec{e_{n}^{l}},\ldots ,\vec{e_{n}^{L_{n}}}\right\}\in R^{L_{n}\times K}$, in which $\overset{⃑}{e_{n}^{l}}=\left\{e_{n}^{l\left\{1\right\}},e_{n}^{l\left\{2\right\}},\ldots ,e_{n}^{l\left\{k\right\}},\ldots ,e_{n}^{l\left\{K\right\}}\right\}{\in R}^{K}$ denotes the K-dimensional feature vector of the *l*th time point.

Any two observed time points in a time-series trajectory can be paired to create two structures that represent their temporal relationships. The two structures are referred to as the $F-G_{\left(i\right)}-D$ and the $F-G_{\left(i\right)}^{`}-D$ structure. Let $t_{1}$ and $t_{2}$ denote the earlier and later time points of the pair, and there are i time intervals between $t_{1}$ and $t_{2}$. The F units corresponding to time points $t_{1}$ and $t_{2}$ are $F_{t_{1}}$ and $F_{t_{2}}$. The $F-G_{\left(i\right)}-D$ structure is the sequential connection of the $F_{t_{1}}$ unit, i copies of the G unit, and then the D unit. This structure can be intuitively interpreted as follows: the system states at time point $t_{1}$ in feature space, undergoing i iterations of forward conversion by G, become the system states at time point $t_{2}$ in feature space, which are then decoded to produce estimated observations. Therefore, the input for this structure is a fixed value presenting the time point $t_{1}$, and the outputs are the estimated observations at time point $t_{2}$. Similarly, the $F-G_{\left(i\right)}^{`}-D$ structure is the sequential connection of the $F_{t_{2}}$ unit, i copies of the $G^{`}$ unit, and then the D unit, which represents the reverse temporal relations as the $F-G_{\left(i\right)}-D$ structure. In both structures, the inputs are fixed values and the outputs are estimated observations. Consequently, the edge weights in these structures can be trained without the need to infer any input data, and the loss function calculated for error propagation requires only reliably observed measurements, which disregards those measurements that are missed or noisy. By this means, BTSTN can effectively learn from partially observed system dynamics without the need for pre-processing.

Therefore, the objectives of BTSTN can be further illustrated as follows: the learning objective is to train the F, G, $G^{`}$, and D units with all possible $F-G_{\left(i\right)}-D$ and $F-G_{\left(i\right)}^{`}-D$ structures from training data. In practice, to avoid computational errors associated with excessively deep networks, we can limit the maximum number of intervals (i) during model training. The reconstruction objective is to estimate complete observations for all time points in the training data using their corresponding F-D structures. The prediction objective is to utilize the trained G, $G^{`}$, and D units to predict future trajectory based on the F units of initial time points in the test data.

For implementation details, please refer to the BTSTN codes, which has been made available at https://github.com/xsh93/BTSTN.

### The p53-Mdm2 ideal dynamic system

p53-Mdm2 is a protein dynamic system in a wide range of species, which features a negative feedback loop (Fig. 1A). We followed reference 48 to simulate this process using two ODEs with explicit time delays. The equations were as follows:


$$\begin{array}{c} \overset{˙}{p}=\alpha -\beta \cdot m\frac{p}{\gamma +p} \end{array}\;\begin{array}{c} \overset{˙}{m}=\psi \cdot p_{\left(t-\tau \right)}-\delta \cdot m \end{array}$$

In this system, transcription factor p53 promotes the synthesis of Mdm2, while Mdm2 facilitates the degradation of p53 by binding to it. The variables *p* and *m* represent the protein levels of p53 and Mdm2, respectively. An explicit time delay *τ* is used to describe the time-delayed regulation. More specifically, the synthesis rate of Mdm2 at current time *t* depends on the protein level of p53 at past time (*t* − *τ*). These system parameters were set, according to the study (48), as follows: *α* = 0.1, *β* = 1, *γ* = 0.01, *ψ* = 0.15, and *δ* = 0.1. Using different values for *τ*, we obtained two oscillation trajectories showing sustained ($\tau =3T_{u}$) or dampened ($\tau =2T_{u}$) behavior ($T_{u}$ is the unit of time). In each group, the levels of p53 and Mdm2 in the biological system are calculated for every $T_{u}$ time unit for subsequent experiments.

### The $\sigma^{28}$-TetR ideal dynamic system

The $\sigma^{28}$-TetR dynamic system is a more complex dynamic system. The transcription factor $\sigma^{28}$ promotes the generation of TetR mRNA, which facilitates the expression of TetR protein. On the other hand, TetR protein inhibits the transcription and expression of $\sigma^{28}$, forming a negative feedback loop. Following the study of Aufinger et al. (50), we simulated this process using four ODEs, in which the delayed regulation effect is achieved by coordination of kinetic parameters instead of using explicit delay *τ*. As shown in Fig. 2A, this system consists of four variables, two of which represent the protein levels of these regulatory proteins (a: $\sigma^{28}$, h: TetR) and two of which represent the mRNA levels of these regulatory proteins ($r_{a}$: $\sigma^{28}$, $r_{h}$: TetR).


$$\begin{array}{c} \overset{˙}{r_{a}}=\alpha_{a}\frac{1}{1+{\left(h/K_{h}\right)}^{n_{h}}}-\left(\delta +\frac{1}{\tau_{m,a}}\right)\cdot r_{a} \end{array}\;\begin{array}{c} \overset{˙}{a}=r_{a}\cdot k_{TL,\;a}-\delta \cdot a \end{array}\;\begin{array}{c} \overset{˙}{r_{h}}=\alpha_{h}\frac{1}{1+{\left(K_{a}/a\right)}^{n_{a}}}-\left(\delta +\frac{1}{\tau_{m,h}}\right)\cdot r_{h} \end{array}\;\begin{array}{c} \overset{˙}{h}=r_{h}\cdot k_{TL,\;h}-\delta \cdot h \end{array}$$

The system parameters were set as follows: $\alpha_{a}=0.1\;nM/s$, $\alpha_{h}=0.1\;nM/s$, $K_{a}=20nM$, $K_{h}=2nM$, $n_{a}=n_{h}=3$, $\tau_{m,a}=\tau_{m,h}=12\;min$, $k_{TL,a}=k_{TL,h}=0.02/s$. Ten sets of initial concentrations for $\sigma^{28}$ and TetR mRNA were used as initial conditions to generate dynamic trajectories, with concentrations ranging from 0 to 1.5 nM (Fig. 2B). In each trajectory, the measurements of the four variables were recorded for every 15 minutes. Eight trajectories were used for model training. The remaining two trajectories were used for the test (Fig. S3).

### Online untargeted metabolomics of succinate fermentation

Cortada-Garcia et al. developed an online untargeted metabolomics technology known as RTMet, which was used to monitor the succinate fermentation process of *E. coli* (33). In this study, samples were automatically collected every 5 minutes from a 5-L bioreactor, and the undiluted fermentation broth was analyzed using a high-resolution Orbitrap mass spectrometer, resulting in 886 different *m*/*z* signals (359 in positive mode and 527 in negative mode). By matching with offline LC-MS signals, 67 metabolites were accurately annotated (Table S9). Throughout the 33-hour fermentation process, 355 available time points were collected. Due to sampling failures and detection abnormalities, each metabolite had some measurements missing. Among the 355 available time points, 17 time points exhibited abnormal fluctuations (1,595–1,695 min and 1,840 min). We masked these data points as missing data. The remaining 338 time points were used for subsequent analyses.

### Linear dynamic systems

We used two ODEs to simulate linear dynamic systems of different behaviors. These behaviors can be obtained by setting appropriate parameters ($\alpha_{1}$, $\alpha_{1}$, $\beta_{1}$, and $\beta_{2}$):


$$\begin{array}{c} \overset{˙}{X}=\alpha_{1}\cdot X+\beta_{1}\cdot Y \end{array}\;\begin{array}{c} \overset{˙}{Y}=\alpha_{2}\cdot X+\beta_{2}\cdot Y \end{array}$$

The parameters ($\alpha_{1}$, $\alpha_{1}$, $\beta_{1}$, and $\beta_{2}$ ) were set to (−3, 2, 2, and −3), (3, 4, −3, and −2), (−0.6, 0.9, 0.9,–0.6), and (0, 2,–3, 0), respectively, to generate the four types of trajectories, i.e., node, focus, saddle point, and center. For each type, nine sets of initial values for *X* and *Y* were used to generate nine trajectories, which were recorded every 0.05 units of time. Among the nine trajectories, eight were used for training and one was used for test.

### Method comparison

The performance of BTSTN was compared with four statistical methods and three deep learning models. We conducted pre-experiments to obtain the optimal hyperparameters for all methods. Each method is implemented as follows:

1. Mean: missing values in a variable are imputed with the average value of the same variable.
2. KNN: Euclidean distance is used to identify the five nearest neighboring time points for a time point with missing measurements based on its known measurements, and a weighted average is computed for the missing measurements (51).
3. MF: the matrix containing missing measurements is decomposed into a low-rank matrix, and a complete matrix is reconstructed using the low-rank matrix, which includes the imputed missing measurements (51).
4. MICE: missing values are first imputed with a range of iterative regression models. Subsequent analyses are performed based on the imputed multiple data sets to obtain reliable estimates (52).
5. BRITS: missing values are imputed using a bidirectional RNN which handles the correlation between multiple missing values in a time series. The RNN hidden size is set to 16 (53).
6. Transformer: a neural network is used to map the input sequence to an output sequence using a self-attention mechanism and an encoder-decoder structure to capture dependencies in the sequence. The parameters of the Transformer model are set to $\;d_{model}$: 264, $d_{ffn}$: 128, number of heads: 4, $d_{v}$: 64, and dropout rate: 0.1 (40).
7. SAITS: a Transformer-based imputation model is used to compute a weighted combination of two diagonally masked self-attention units (47). For analyses of the ideal system data sets, the model parameters are set to $\;d_{model}$: 264, $d_{ffn}$: 128, number of heads: 4, $d_{v}$: 64, and dropout rate: 0.1. For analysis of the succinate fermentation data set, the model parameters are set to $\;d_{model}$: 256, $d_{ffn}$: 512, number of heads: 4, $d_{v}$: 64, and dropout rate: 0.2.
8. BTSTN: for analyses of the ideal system data sets, the output dimension of each F unit is set to 16; the hidden layer dimension of the G or $G^{`}$ unit is set to 32; the number of hidden layers is set to 2; and the dropout rate is set to 0.1. For analysis of the succinate fermentation data set, a total of 338 F units with an output dimension of 256 were used. The input and output dimensions of the G or $G^{`}$ unit are set to the same value of 256; the numbers of hidden layer and dimension are set to 4 and 256, respectively; and the output dimension of the D unit is set to 67, which is equal to the number of variables in the observed data. The dropout rate for all units is set to 0.2.

The Python package fancyimpute (55) was used to implement the mean, KNN, MF, and MICE methods, and the Python package pypots (56) was used to implement BRITS, Transformer, and SAITS models. All the deep learning models were implemented with the PyTorch framework supporting MPS acceleration and were trained on an Apple M1 processor using the Adam optimizer. The learning rate was uniformly set to 0.001, and the batch size was uniformly set to 128. In addition, an early stopping strategy was used, which terminated training after 30 epochs if no decrease in MAE was observed.

### Performance evaluation

To evaluate the performance of methods, we randomly masked a portion of the observed data and used an artificial mask vector $I=\left\{i_{1},i_{2},\ldots ,i_{n},\ldots i_{N}\right\}$ to present this missing information, in which $I_{n}\in {\left\{0,1\right\}}^{Ln\times D}$ is the mask for trajectory $S_{n}$:


$$\begin{array}{c} I_{n}^{l\left\{d\right\}}=\left\{ \begin{array}{ll} 1,\;\; & if\;x_{n}^{l\left\{d\right\}}\;is\;masked\\ 0,\;\; & otherwise \end{array}\right. \end{array}$$

The performance of methods was quantified with error measures using three metrics, i.e., MAE, RMSE, and MRE, which were calculated as follows:


$$\begin{array}{c} MAE=\frac{\sum_{n=1}^{N}\sum_{l=1}^{L_{n}}\sum_{d}^{D}{\left|\left(estimation-target\right)⨀mask\right|}_{n}^{l\left\{d\right\}}}{\sum_{n=1}^{N}\sum_{l=1}^{L_{n}}\sum_{d=1}^{D}mask_{n}^{l\left\{d\right\}}}\# \end{array}\;\begin{array}{c} RMSE=\sqrt{\frac{\sum_{n=1}^{N}\sum_{l=1}^{L_{n}}\sum_{d}^{D}{\left|{\left(\left(estimation-target\right)⨀mask\right)}^{2}\right|}_{n}^{l\left\{d\right\}}}{\sum_{n=1}^{N}\sum_{l=1}^{L_{n}}\sum_{d=1}^{D}mask_{n}^{l\left\{d\right\}}}}\# \end{array}\;\begin{array}{c} MRE=\frac{\sum_{n=1}^{N}\sum_{l=1}^{L_{n}}\sum_{d}^{D}{\left|\left(estimation-target\right)⨀mask\right|}_{n}^{l\left\{d\right\}}}{\sum_{n=1}^{N}\sum_{l=1}^{L_{n}}\sum_{d}^{D}{\left|target⨀mask\right|}_{n}^{l\left\{d\right\}}} \end{array}$$

where “estimation” is the model-estimated values and “target” is the ground truth.

The correlation between the predicted trajectory and the ground truth is evaluated with the Pearson correlation coefficient, which is calculated as follows:


$$\begin{array}{c} \rho_{T,\;P}=\frac{cov\left(T,\;P\right)}{\sigma_{T}\sigma_{P}}=\frac{\sum \left(T-\overset{-}{T}\right)\left(P-\overset{-}{P}\right)}{\sqrt{\sum {\left(T-\overset{-}{T}\right)}^{2}\sum {\left(P-\overset{-}{P}\right)}^{2}}} \end{array}$$

where “cov” is the covariance and σ is the standard deviation; *T* and $\overset{-}{T}$ are the ground truth and its respective mean value, while *P* and $\overset{-}{P}$ are the predicted trajectory and its respective mean value.

All the experiments were repeated three times to obtain the mean value and respective confidence intervals for each estimated measurement. Each repetition involved random initialization of model parameters, including featurizer, generator, and decoder.

## Data Availability

The Bidirectional Time-series State Transfer Network was developed with Python and Pytorch framework. All the source codes and data sets are available at https://github.com/xsh93/BTSTN.

## SUPPLEMENTAL MATERIAL

Supplemental material msystems.00697-24-s0001.docx BTSTN implementation, Figures S1-S7, and Tables S1-S11.

ASM does not own the copyrights to Supplemental Material that may be linked to, or accessed through, an article. The authors have granted ASM a non-exclusive, world-wide license to publish the Supplemental Material files. Please contact the corresponding author directly for reuse.
