# Supplementary material for: Learning metabolic dynamics from irregular observations by Bidirectional Time-Series State Transfer Network
Source: mSystems. 2024 Jul 26;9(8):e00697-24. doi: 10.1128/msystems.00697-24 (PMC11334518; doi:10.1128/msystems.00697-24)
Supplement: Supplemental material — BTSTN implementation, Figures S1-S7, and Tables S1-S11. [file msystems.00697-24-s0001.docx]

**Supplemental Materials for**

**Learning Metabolic Dynamics from Irregular Observations
by Bidirectional Time-series State Transfer Network**

Shaohua Xu^a, b, #^, Ting Xu^a, #^, Yuping Yang^a^, Xin Chen^a, b, *^

^a^ School of Basic Medical Sciences and the First Affiliated Hospital Department of Radiation Oncology, Zhejiang University School of Medicine, Hangzhou, 310058, China

^b^ Zhejiang Provincial Key Laboratory for Microbial Biochemistry and Metabolic Engineering, Hangzhou, 310058, China

^#^ These authors made equal contributions.

^*^ Correspondence to [xinchen@zju.edu.cn](mailto:xinchen@zju.edu.cn)

**This file includes:**

- BTSTN Implementation
- Fig. S1-S7
- Table S1-S11.

**BTSTN Implementation**

As illustrated in the “Materials and Methods” section, the training of BTSTN involves bidirectional training structures supervised by paired time points in a time-series trajectory. This process is computationally intensive. To streamline the training process for parallel computation, we developed the following implementation.

**1. Data preparation**

First, three matrices are used in model training: a One-hot matrix, a Countdown matrix, and an Observation matrix (Fig. 1B). The One-hot matrix marks all available time points. It is prepared with each row representing the starting time point of a pair, by assigning a random input value (default 1) to a specific column and 0 to all others. The Countdown matrix tracks the generation times (forward or backward) with each row representing the time interval between the starting and ending timepoint of a pair. The Observation matrix is prepared with each row representing the observations of the ending time point of a pair, which may contain missing values.

**2. Model training**

During model training, *F* units first convert the One-hot matrix into a feature matrix for all time points. Subsequently, the feature matrix undergoes several rounds of generation by Generators (*G* or *G’* units). After each round, the count value in each row of the Countdown matrix is subtracted by 1 until the value reaches 0. Simultaneously, two Boolean matrices are generated to indicate the completion status of the generation process. Upon all the count value reaches 0, the output matrix is decoded as the predicted values by the *D* unit. In addition, we introduced the residual modules in each generation step to enhance the expressive ability of deep networks. The loss calculated between the predicted values and the Observation matrix is used for updating network parameters (Fig. 1C).

All these procedures have been implemented in the Python package (BTSTN). The latest source codes are available at <https://github.com/xsh93/BTSTN>.

| 1. **Algorithm 1** BTSTN Training |
| --- |
| 1. **Input:** The set of training data for the current batch ; 2. One-hot matrix: ; 3. Countdown matrix: ; 4. Observation matrix: ; 5. missing matrix: ; 6. The initialized parameters: Featurizer $\theta_{F}$, forward Generator $\theta_{G}$, backward Generator $\theta_{G'}$, and Decoder ; 7. **Output:** Trained parameters $\theta_{F}$, $\theta_{G}$, $\theta_{G'}$, $\theta_{D}$; learned temporal features $e_{n}\in\mathbb{R}^{L_{n}\times K}$ $e_{n}\leftarrow F\left( h_{n} \right)$ ${c1}_{n}\leftarrow0_{L_{n}}$ ${c2}_{n}\leftarrow0_{L_{n}}$ 8. **while** not converged **do** 9. **for** each time point $t_{n}^{l}\in t_{n}$ **do** 10. **while** $c_{n}^{l}>=0$ **do** 11. **if** $c_{n}^{l}>0$ **then** ${c1}_{n}^{l}\leftarrow0$ ${c2}_{n}^{t}\leftarrow1$ 14. **else** ${c1}_{n}^{l}\leftarrow1$ ${c2}_{n}^{t}\leftarrow0$ 17. **end if** $d\leftarrow{d+e}_{n}^{l}\cdot{c1}_{n}^{l}$ 19. **If** generated in forward direction **then** $e_{n}^{l}\leftarrow(G\left( e_{n}^{l} \right)+e_{n}^{l})\cdot{c2}_{n}^{l}$ 21. **else** $e_{n}^{l}\leftarrow(G'\left( e_{n}^{l} \right)+e_{n}^{l})\cdot{c2}_{n}^{l}$ 23. **end if** $c_{n}^{l}\leftarrow c_{n}^{l}-1$ 25. **end while** 26. **end for** 27. **end while** |

**FIG S1** Pseudocode for the BTSTN algorithm. For input representation, $s_{n}$ denotes the *n*-th time-series trajectory of observation data. $L_{n}$ and *D* denote the number of observed time points and variables in $s_{n}$, respectively. For output representation, $e_{n}$ denotes the *n*-th trajectory in the feature space, corresponding to $s_{n}$. And *K* denotes the number of feature dimensions in $e_{n}$. For training representation, ${c1}_{n}$ and ${c2}_{n}$ are two paired matrices indicating the completion status of the generation process for all time points $t_{n}$. When $c_{n}^{l}$ reaches 0, it indicates the generation process for $t_{n}^{l}$ is completed, and the corresponding ${c1}_{n}^{l}$ will be assigned to 1, and ${c2}_{n}^{l}$ will be assigned to 0.


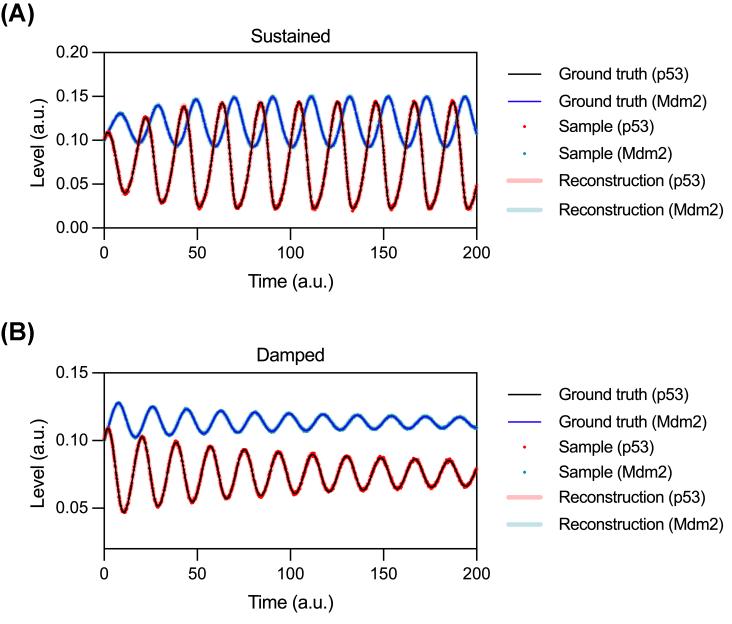


**FIG S2** Reconstruction of the p53-Mdm2 dynamic systems representing the sustained (A) and damped (B) oscillations. The training data masked with a missing rate of 30% and Gaussian noise (standard deviation 0.01) from the ground truth (black and blue curves) are presented in red and green dots. The light red and light green curves are the reconstructed trajectories for the p53 and Mdm2 measurements, respectively.


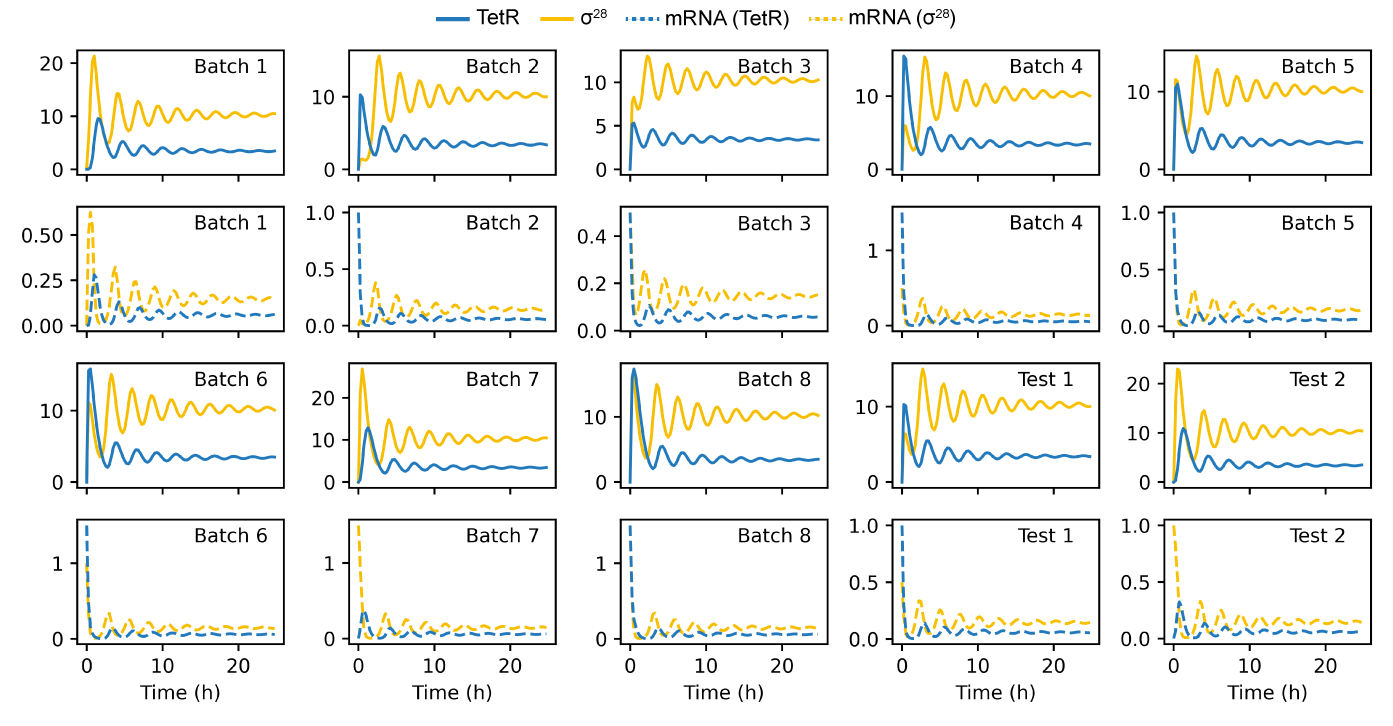


**FIG S3** Simulated time-series trajectories of the $\sigma^{28}$-TetR dynamic system. Eight batches (Batch 1-8) are used for training and two batches (Test 1-2) for test.


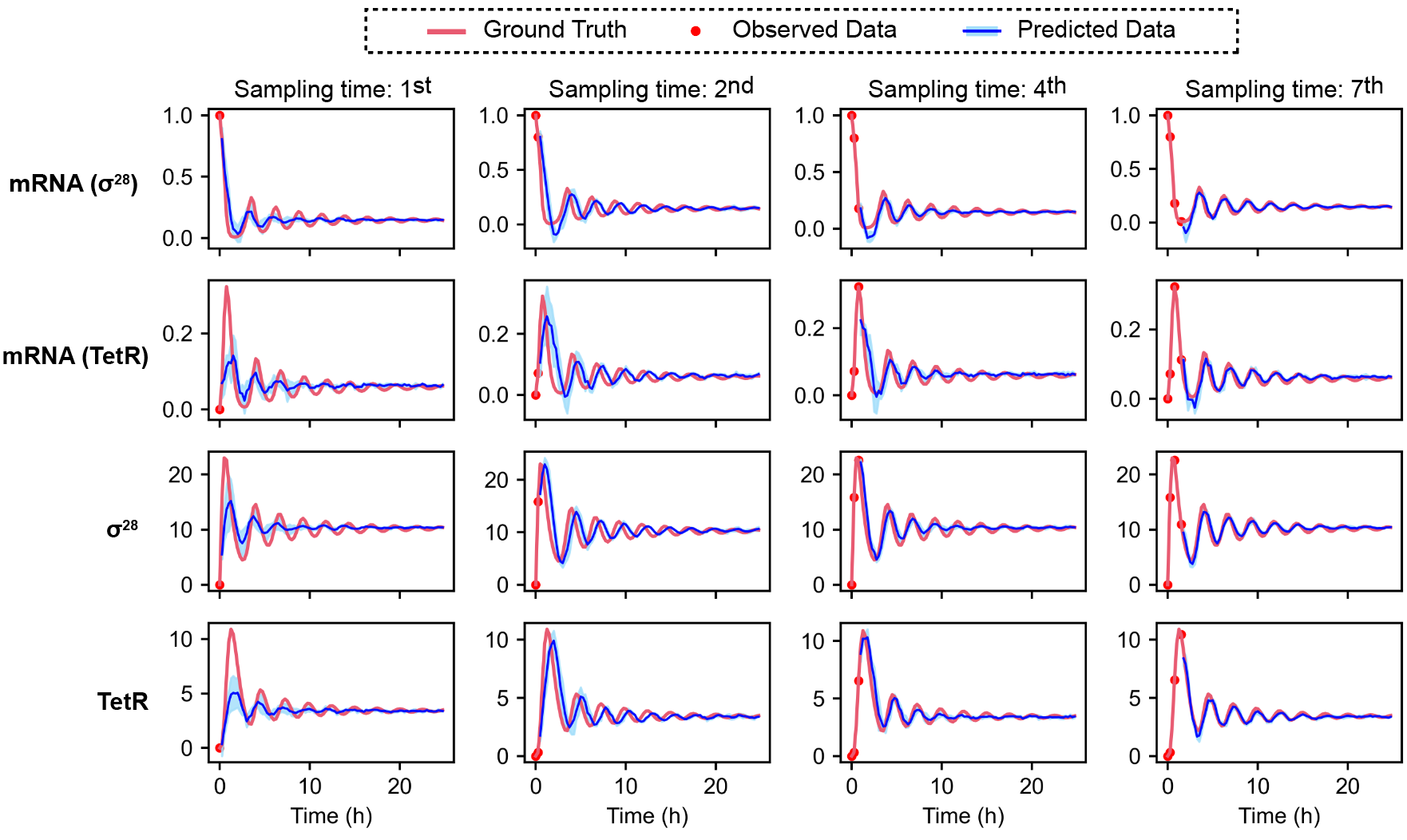


**FIG S4** Prediction (blue curves) of the future trajectories of the Test 2 dataset from the $\sigma^{28}$-TetR dynamic system based on the observations of four discontinuous time points, i.e., the 1^st^, 2^nd^, 4^th^, 7^th^ time points. The shaded region (light blue) corresponds to the $\pm$one standard deviation and the red dots represent observed data from ground truth (red curves).


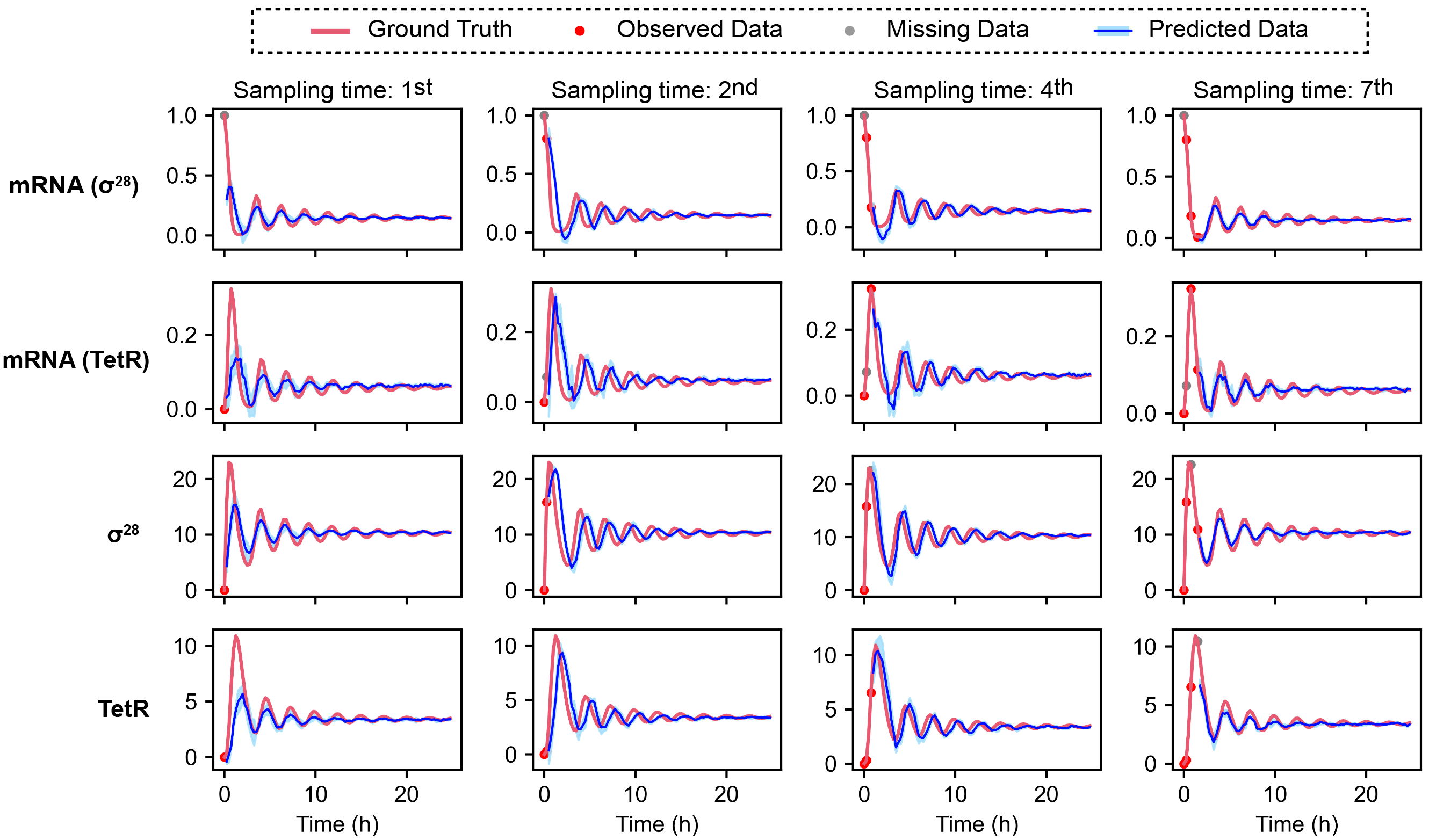


**FIG S5** Prediction (blue curves) of the future trajectories of the Test 2 dataset from the $\sigma^{28}$-TetR dynamic system based on the observations containing missing values of four discontinuous time points, i.e., the 1^st^, 2^nd^, 4^th^, 7^th^ time points. The shaded region (light blue) corresponds to the $\pm$one standard deviation. The red and grey dots represent observed and missing data from ground truth (red curves), respectively.


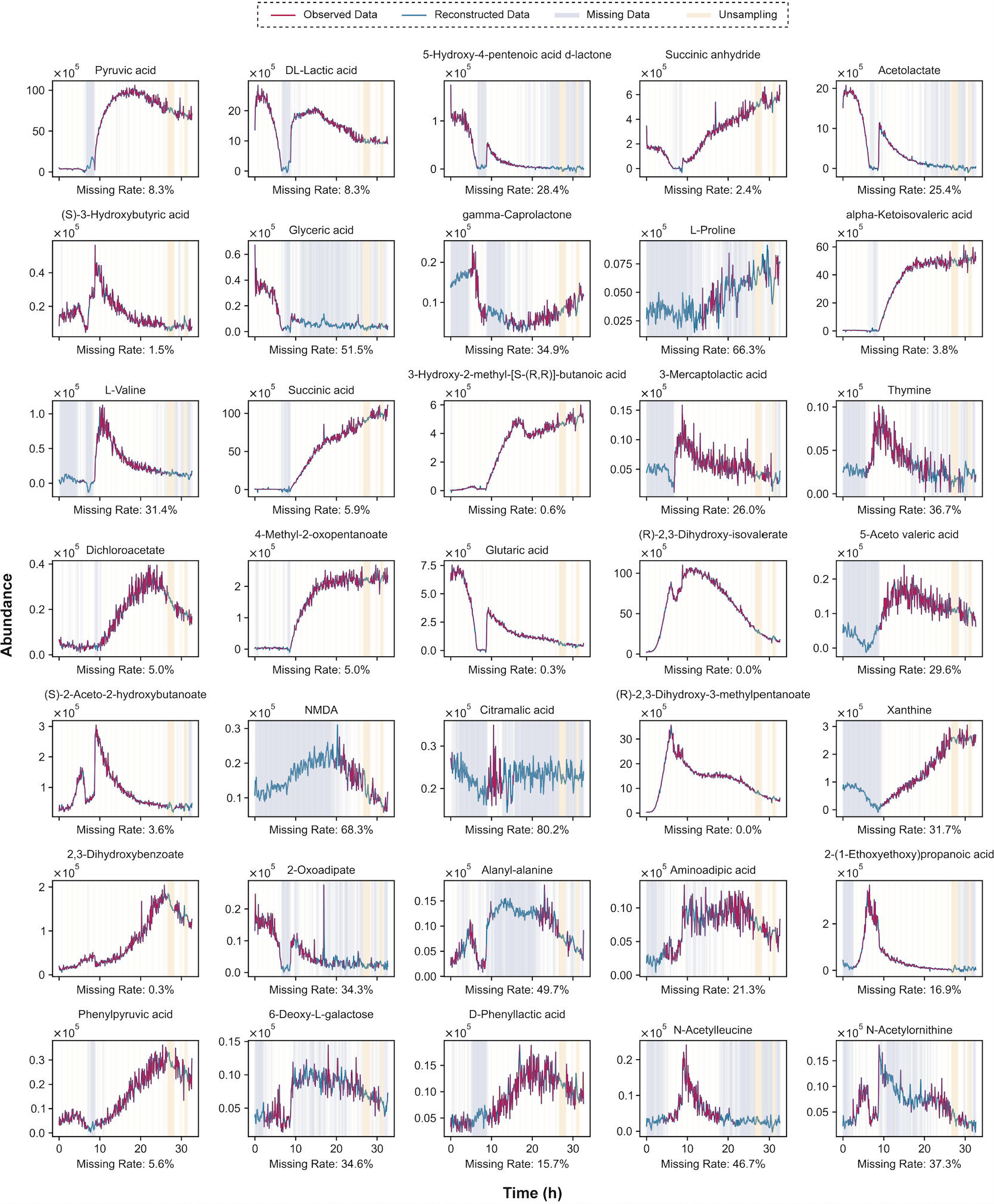


**FIG S6** Reconstruction of the trajectories of 35 metabolites (dark green curves) from the succinate fermentation dataset with BTSTN based on the available observations (dark red curves). The light yellow and light blue areas indicate the unsampled time points and the missing values of corresponding metabolites, respectively.


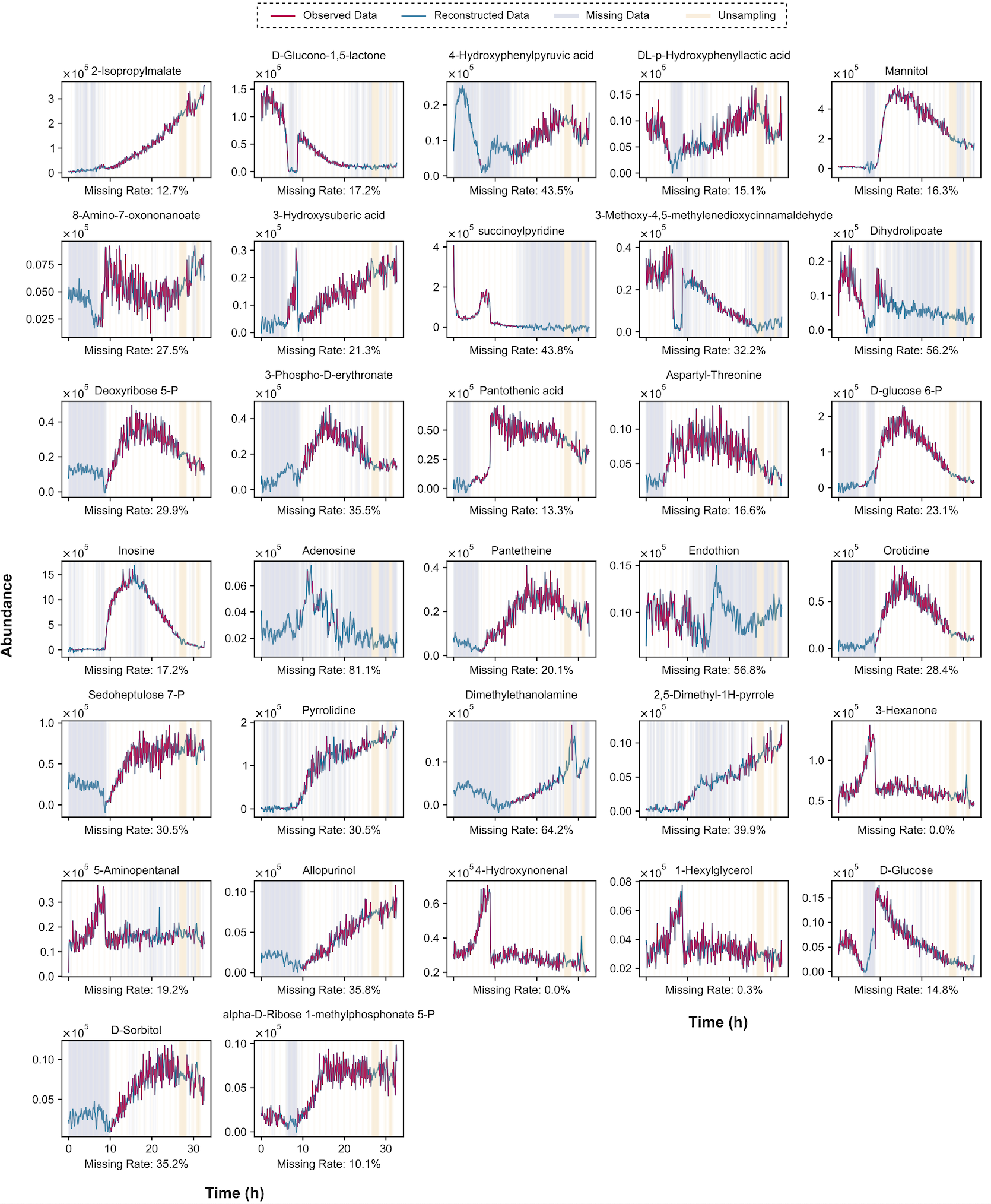


**FIG S7** Reconstruction of the trajectories of 32 metabolites (dark green curves) from the succinate fermentation dataset with BTSTN based on the available observations (dark red curves). The light yellow and light blue areas indicate the unsampled time points and the missing values of corresponding metabolites, respectively.

**TABLE S1** Reconstruction performance of BTSTN on the sustained and damped p53-Mdm2 datasets with different missing distributions. Metrics (mean ± SD, 10^-2) are reported in the order of MAE/RMSE/MRE.

| Missing Distribution | Sustained - p53 | | |  | Sustained - Mdm2 | | |
| --- | --- | --- | --- | --- | --- | --- | --- |
|  | MAE | RMSE | MRE | | MAE | RMSE | MRE |
| Non-missing | 0.0518 ± 0.0091 | 0.0669 ± 0.0122 | 66.14% ± 11.66% | | 0.0251 ± 0.0028 | 0.0322 ± 0.0037 | 15.43% ± 7.34% |
| p53 | 0.1335 ± 0.0281 | 0.1632 ± 0.0314 | 170.34% ± 35.90% | | 0.0314 ± 0.0051 | 0.0393 ± 0.0056 | 26.45% ± 4.27% |
| Mdm2 | 0.0608 ± 0.0111 | 0.0768 ± 0.0135 | 77.55% ± 14.13% | | 0.0413 ± 0.0044 | 0.0524 ± 0.0058 | 34.87% ± 3.74% |
| p53 / Mdm2 | 0.0808 ± 0.0133 | 0.1023 ± 0.0164 | 103.13% ± 16.96% | | 0.0311 ± 0.0037 | 0.0400 ± 0.0052 | 26.23% ± 3.12% |
|  |  | | |  |  | | |
| Missing Distribution | Damped - p53 | | |  | Damped - Mdm2 | | |
|  | MAE | RMSE | MRE | | MAE | RMSE | MRE |
| Non-missing | 0.0224 ± 0.0020 | 0.0292 ± 0.0027 | 29.55% ± 2.69% | | 0.0092 ± 0.0013 | 0.0121 ± 0.0017 | 8.11% ± 1.17% |
| p53 | 0.0500 ± 0.0039 | 0.0651 ± 0.0068 | 65.96% ± 5.08% | | 0.0140 ± 0.0044 | 0.0181 ± 0.0051 | 12.32% ± 3.85% |
| Mdm2 | 0.0240 ± 0.0018 | 0.0311 ± 0.0022 | 31.60% ± 2.36% | | 0.0155 ± 0.0032 | 0.0203 ± 0.0040 | 13.64% ± 2.77% |
| p53 / Mdm2 | 0.0236 ± 0.0026 | 0.0306 ± 0.0036 | 31.15% ± 3.45% | | 0.0105 ± 0.0011 | 0.0139 ± 0.0017 | 9.21% ± 1.00% |

**TABLE S2** Reconstruction performance of BTSTN on the sustained and damped p53-Mdm2 datasets with Gaussian noise (standard deviation 0.01) across different missing rates from 10% to 80%. Metrics (mean ± SD, 10^-2) are reported in the order of MAE/RMSE/MRE.

| Missing Rate | Dataset: Sustained | | |  | Dataset: Damped | | |
| --- | --- | --- | --- | --- | --- | --- | --- |
|  | MAE | RMSE | MRE | | MAE | RMSE | MRE |
| 10% | 0.0886 ± 0.0061 | 0.1177 ± 0.0091 | 90.02% ± 6.23% | | 0.0425 ± 0.0032 | 0.0584 ± 0.0061 | 44.84% ± 3.38% |
| 20% | 0.0844 ± 0.0097 | 0.1115 ± 0.0148 | 85.71% ± 9.89% | | 0.0381 ± 0.0036 | 0.0526 ± 0.0046 | 40.19% ± 3.82% |
| 30% | 0.0936 ± 0.0064 | 0.1273 ± 0.0088 | 95.03% ± 6.50% | | 0.0417 ± 0.0037 | 0.0575 ± 0.0059 | 44.00% ± 3.87% |
| 40% | 0.0931 ± 0.0012 | 0.1257 ± 0.0020 | 94.51% ± 1.26% | | 0.0438 ± 0.0046 | 0.0590 ± 0.0073 | 46.17% ± 4.90% |
| 50% | 0.0923 ± 0.0058 | 0.1226 ± 0.0076 | 93.75% ± 5.86% | | 0.0439 ± 0.0015 | 0.0585 ± 0.0009 | 46.29% ± 1.55% |
| 60% | 0.0992 ± 0.0016 | 0.1324 ± 0.0033 | 100.74% ± 1.58% | | 0.0458 ± 0.0034 | 0.0614 ± 0.0054 | 48.29% ± 3.58% |
| 70% | 0.1028 ± 0.0061 | 0.1380 ± 0.0080 | 104.37% ± 6.23% | | 0.0463 ± 0.0040 | 0.0627 ± 0.0054 | 48.81% ± 4.23% |
| 80% | 0.1617 ± 0.0126 | 0.2316 ± 0.0125 | 164.19% ± 12.76% | | 0.0574 ± 0.0042 | 0.0786 ± 0.0053 | 60.52% ± 4.45% |

**TABLE S3** Extrapolation performance of BTSTN on the Test 1 and 2 datasets of the $\sigma^{28}$-TetR datasets across different missing rates from 10% to 80%. Metrics (mean ± SD) are reported in the order of MAE/RMSE/MRE.

| Missing Rate | Eval 1 | | |  | Eval 2 | | |
| --- | --- | --- | --- | --- | --- | --- | --- |
|  | MAE | RMSE | MRE | | MAE | RMSE | MRE |
| 10% | 0.1732 ± 0.0111 | 0.4616 ± 0.0219 | 5.02% ± 0.32% | | 0.1582 ± 0.0033 | 0.4419 ± 0.0201 | 4.31% ± 0.09% |
| 20% | 0.1645 ± 0.0111 | 0.4617 ± 0.0409 | 4.77% ± 0.32% | | 0.1462 ± 0.0005 | 0.4162 ± 0.0019 | 4.62% ± 0.02% |
| 30% | 0.1643 ± 0.0051 | 0.4310 ± 0.0081 | 4.77% ± 0.15% | | 0.1519 ± 0.0052 | 0.4034 ± 0.0165 | 4.37% ± 0.15% |
| 40% | 0.1542 ± 0.0044 | 0.4060 ± 0.0238 | 4.47% ± 0.13% | | 0.1554 ± 0.0072 | 0.3681 ± 0.0198 | 4.47% ± 0.21% |
| 50% | 0.1639 ± 0.0026 | 0.4318 ± 0.0170 | 4.76% ± 0.08% | | 0.1562 ± 0.0131 | 0.4034 ± 0.0361 | 4.53% ± 0.38% |
| 60% | 0.1851 ± 0.0047 | 0.6549 ± 0.1247 | 5.37% ± 0.14% | | 0.1664 ± 0.0076 | 0.4469 ± 0.0082 | 4.80% ± 0.22% |
| 70% | 0.1851 ± 0.0072 | 0.7922 ± 0.0109 | 5.37% ± 0.21% | | 0.1964 ± 0.0124 | 0.6151 ± 0.1074 | 5.53% ± 0.35% |
| 80% | 0.2159 ± 0.0112 | 0.7955 ± 0.1425 | 6.26% ± 0.32% | | 0.2311 ± 0.0133 | 0.6658 ± 0.0352 | 6.46% ± 0.37% |

**TABLE S4** Comparison of interpolation performance among various methods on the $\sigma^{28}$-TetR datasets across different missing rates from 10% to 80%. Metrics (mean ± SD) are reported in the order of MAE/RMSE/MRE.

| Method | Missing Rate: 10% | | |  | Missing Rate: 20% | | | |  | Missing Rate: 30% | | |
| --- | --- | --- | --- | --- | --- | --- | --- | --- | --- | --- | --- | --- |
|  | MAE | RMSE | MRE (%) | | MAE | RMSE | | MRE (%) | | MAE | RMSE | MRE (%) | |
| Mean* | 0.5738 | 1.3303 | 16.18 | | 0.6666 | 1.7146 | | 18.02 | | 0.6792 | 1.7279 | 18.24 | |
| KNN* | 0.4537 | 1.2269 | 12.79 | | 0.5757 | 1.6188 | | 15.56 | | 0.6880 | 1.9502 | 18.47 | |
| MF* | 0.5717 | 1.3094 | 16.12 | | 0.7066 | 1.7301 | | 19.10 | | 0.7154 | 1.7356 | 19.21 | |
| MICE* | 0.6203 | 4.1858 | 17.49 | | 0.7342 | 4.4051 | | 19.85 | | 0.6565 | 3.3424 | 17.63 | |
| BRITS | 0.3450 ± 0.0123 | 0.9255 ± 0.0339 | 9.73 ± 0.35 | | 0.4435 ± 0.0246 | 1.4057 ± 0.0442 | | 11.99 ± 0.67 | | 0.5440 ± 0.0287 | 1.5287 ± 0.0328 | 14.61 ± 0.77 | |
| Transformer | 0.4766 ± 0.0082 | 1.0922 ± 0.0291 | 13.44 ± 0.23 | | 0.5734 ± 0.0038 | 1.4973 ± 0.0417 | | 15.50 ± 0.10 | | 0.5899 ± 0.0044 | 1.5752 ± 0.0085 | 15.84 ± 0.12 | |
| SAITS | 0.4414 ± 0.0191 | 0.9996 ± 0.0511 | 12.44 ± 0.54 | | 0.4630 ± 0.0553 | 1.1880 ± 0.1705 | | 12.51 ± 1.50 | | 0.5071 ± 0.0327 | 1.3559 ± 0.1061 | 13.61 ± 0.88 | |
| BTSTN | **0.1761 ± 0.0047** | **0.4938 ± 0.0223** | **4.96 ± 0.13** | | **0.2391 ± 0.0020** | **0.8731 ± 0.0630** | | **6.46 ± 0.05** | | **0.2442 ± 0.0066** | **0.8701 ± 0.0237** | **6.56 ± 0.18** | |
| Method | Missing Rate: 40% | | |  | Missing Rate: 50% | | | |  | Missing Rate: 60% | | | |
|  | MAE | RMSE | MRE (%) | | MAE | RMSE | | MRE (%) | | MAE | RMSE | MRE (%) | |
| Mean* | 0.6309 | 1.5895 | 17.36 | | 0.6329 | 1.6338 | | 17.63 | | 0.6194 | 1.6061 | 17.35 | |
| KNN* | 0.6850 | 1.9446 | 18.85 | | 0.8928 | 2.4624 | | 24.87 | | 1.1886 | 2.9527 | 33.30 | |
| MF* | 0.6739 | 1.5809 | 18.55 | | 0.7005 | 1.6436 | | 19.51 | | 0.6985 | 1.6236 | 19.57 | |
| MICE* | 0.6304 | 2.4277 | 17.35 | | 0.5488 | 1.6320 | | 15.29 | | 0.5695 | 1.7099 | 15.95 | |
| BRITS | 0.5810 ± 0.0228 | 1.4952 ± 0.0114 | 15.99 ± 0.63 | | 0.5970 ± 0.0174 | 1.5591 ± 0.0177 | | 16.63 ± 0.48 | | 0.5739 ± 0.0156 | 1.5199 ± 0.0082 | 16.08 ± 0.44 | |
| Transformer | 0.5541 ± 0.0113 | 1.4484 ± 0.0252 | 15.25 ± 0.31 | | 0.5611 ± 0.0066 | 1.5194 ± 0.0456 | | 15.63 ± 0.18 | | 0.5616 ± 0.0018 | 1.4636 ± 0.0180 | 15.73 ± 0.05 | |
| SAITS | 0.5042 ± 0.0221 | 1.3026 ± 0.0840 | 13.87 ± 0.61 | | 0.5008 ± 0.0088 | 1.3548 ± 0.0248 | | 13.95 ± 0.25 | | 0.5363 ± 0.0154 | 1.4684 ± 0.0741 | 15.02 ± 0.43 | |
| BTSTN | **0.2610 ± 0.0076** | **0.8603 ± 0.0296** | **7.18 ± 0.21** | | **0.2671 ± 0.0042** | **1.1221 ± 0.0147** | | **7.44 ± 0.12** | | **0.2929 ± 0.0045** | **1.1177 ± 0.0308** | **8.20 ± 0.13** | |
| Method | Missing Rate: 70% | | |  | Missing Rate: 80% | |  | | |  | | | |
|  | MAE | RMSE | MRE (%) | | MAE | RMSE | | MRE (%) | |  |  |  | |
| Mean* | 0.6083 | 1.5426 | 17.30 | | 0.6207 | 1.5185 | | 17.56 | |  |  |  | |
| KNN* | 1.5509 | 3.4660 | 44.11 | | 2.1190 | 4.1738 | | 59.95 | |  |  |  | |
| MF* | 0.7028 | 1.5646 | 19.99 | | 0.7672 | 1.5681 | | 21.70 | |  |  |  | |
| MICE* | 0.6403 | 1.7543 | 18.21 | | 0.6187 | 1.5231 | | 17.50 | |  |  |  | |
| BRITS | 0.5438 ± 0.0171 | 1.4582 ± 0.0168 | 15.46 ± 0.49 | | 0.5958 ± 0.0207 | 1.4954 ± 0.0134 | | 16.86 ± 0.59 | |  |  |  | |
| Transformer | 0.5795 ± 0.0148 | 1.4481 ± 0.0142 | 16.48 ± 0.42 | | 0.6380 ± 0.0218 | 1.5391 ± 0.0503 | | 18.05 ± 0.62 | |  |  |  | |
| SAITS | 0.5295 ± 0.0141 | 1.4126 ± 0.0489 | 15.06 ± 0.40 | | 0.5460 ± 0.0067 | 1.4006 ± 0.0691 | | 15.45 ± 0.19 | |  |  |  | |
| BTSTN | **0.3029 ± 0.0074** | **1.2230 ± 0.0359** | **8.61 ± 0.21** | | **0.3337 ± 0.0072** | **1.1835 ± 0.0339** | | **9.44 ± 0.21** | |  |  |  | |

Note: **Values** in bold are the best and values underlined are the second best. Statistical methods labeled with * performed only once.

**TABLE S5** Comparison of extrapolation performance among various methods on the $\sigma^{28}$-TetR datasets across different missing rates from 10% to 80%. Metrics (mean ± SD) are reported in the order of MAE/RMSE/MRE.

| Method | Missing Rate: 10% | | |  | Missing Rate: 20% | | | |  | Missing Rate: 30% | | |
| --- | --- | --- | --- | --- | --- | --- | --- | --- | --- | --- | --- | --- |
|  | MAE | RMSE | MRE (%) | | MAE | RMSE | | MRE (%) | | MAE | RMSE | MRE (%) | |
| Mean* | 0.5122 | 1.1479 | 13.95 | | 0.5028 | 1.170 | | 15.89 | | 0.5162 | 1.1208 | 14.84 | |
| KNN* | 0.5371 | 1.3195 | 14.63 | | 0.6354 | 1.4609 | | 20.08 | | 0.5752 | 1.2845 | 16.53 | |
| MF* | 0.5297 | 1.1480 | 14.43 | | 0.5557 | 1.2474 | | 17.56 | | 0.5841 | 1.2806 | 16.79 | |
| MICE* | 0.3580 | 0.9547 | 9.75 | | 0.4288 | 1.1100 | | 13.55 | | 0.5630 | 2.0464 | 16.18 | |
| BRITS | 0.3299 ± 0.0192 | 0.9748 ± 0.0149 | 8.99 ± 0.52 | | 0.3807 ± 0.0314 | 1.0045 ± 0.0435 | | 12.03 ± 0.99 | | 0.4602 ± 0.0219 | 1.0651 ± 0.0399 | 13.23 ± 0.63 | |
| Transformer | 0.4721 ± 0.0131 | 1.0391 ± 0.0148 | 12.86 ± 0.36 | | 0.4161 ± 0.0158 | 0.8978 ± 0.0186 | | 13.15 ± 0.50 | | 0.4378 ± 0.0070 | 0.9129 ± 0.0172 | 12.59 ± 0.20 | |
| SAITS | 0.3935 ± 0.0672 | 0.8163 ± 0.1268 | 10.72 ± 1.83 | | 0.3817 ± 0.0520 | 0.7920 ± 0.1290 | | 12.06 ± 1.64 | | 0.4336 ± 0.0381 | 0.9244 ± 0.0343 | 12.46 ± 1.10 | |
| BTSTN | **0.1306 ± 0.0094** | **0.4038 ± 0.0198** | **3.56 ± 0.26** | | **0.1413 ± 0.0184** | **0.3725 ± 0.0407** | | **4.46 ± 0.58** | | **0.1501 ± 0.0089** | **0.3959 ± 0.0085** | **4.31 ± 0.26** | |
| Method | Missing Rate: 40% | | |  | Missing Rate: 50% | | | |  | Missing Rate: 60% | | | |
|  | MAE | RMSE | MRE (%) | | MAE | RMSE | | MRE (%) | | MAE | RMSE | MRE (%) | |
| Mean* | 0.5467 | 1.1721 | 15.73 | | 0.5400 | 1.2198 | | 15.66 | | 0.5304 | 1.1659 | 15.29 | |
| KNN* | 0.7304 | 1.8253 | 21.01 | | 0.8919 | 2.2730 | | 25.87 | | 1.1749 | 2.8373 | 33.88 | |
| MF* | 0.5908 | 1.1885 | 17.00 | | 0.6052 | 1.2444 | | 17.55 | | 0.6346 | 1.2688 | 18.30 | |
| MICE* | 0.5112 | 1.3813 | 14.71 | | 0.5488 | 1.2586 | | 15.92 | | 0.6227 | 1.4153 | 17.95 | |
| BRITS | 0.4930 ± 0.0091 | 1.1381 ± 0.0087 | 14.18 ± 0.26 | | 0.5234 ± 0.0480 | 1.1946 ± 0.0593 | | 15.18 ± 1.39 | | 0.5388 ± 0.0202 | 1.1859 ± 0.0192 | 15.53 ± 0.58 | |
| Transformer | 0.4677 ± 0.0100 | 1.1125 ± 0.0157 | 13.45 ± 0.29 | | 0.4972 ± 0.0275 | 1.1018 ± 0.0323 | | 14.42 ± 0.80 | | 0.5140 ± 0.0203 | 1.1046 ± 0.0170 | 14.82 ± 0.59 | |
| SAITS | 0.4462 ± 0.0283 | 1.1088 ± 0.0430 | 12.83 ± 0.81 | | 0.4948 ± 0.0191 | 1.1411 ± 0.0565 | | 14.35 ± 0.55 | | 0.5052 ± 0.0148 | 1.1349 ± 0.0226 | 14.57 ± 0.43 | |
| BTSTN | **0.1547 ± 0.0014** | **0.4071 ± 0.0532** | **4.45 ± 0.04** | | **0.1609 ± 0.0058** | **0.4014 ± 0.0373** | | **4.67 ± 0.17** | | **0.1958 ± 0.0191** | **0.5018 ± 0.0493** | **5.65 ± 0.55** | |
| Method | Missing Rate: 70% | | |  | Missing Rate: 80% | |  | | |  | | | |
|  | MAE | RMSE | MRE (%) | | MAE | RMSE | | MRE (%) | |  |  |  | |
| Mean* | 0.5742 | 1.3148 | 16.18 | | 0.6854 | 1.4653 | | 19.16 | |  |  |  | |
| KNN* | 1.4837 | 3.2530 | 41.81 | | 1.9660 | 3.9810 | | 54.96 | |  |  |  | |
| MF* | 0.6933 | 1.3788 | 19.54 | | 0.8585 | 1.5787 | | 24.00 | |  |  |  | |
| MICE* | 0.6158 | 1.4230 | 17.35 | | 0.6858 | 1.4654 | | 19.17 | |  |  |  | |
| BRITS | 0.5316 ±0.0144 | 1.2876 ± 0.0111 | 14.98 ± 0.41 | | 0.5617 ± 0.0079 | 1.4553 ± 0.0148 | | 15.70 ± 0.22 | |  |  |  | |
| Transformer | 0.5427 ± 0.0116 | 1.3419 ± 0.0374 | 15.29 ± 0.33 | | 0.6282 ± 0.0531 | 1.5716 ± 0.0443 | | 17.56 ± 1.48 | |  |  |  | |
| SAITS | 0.5292 ± 0.0121 | 1.3265 ± 0.0020 | 14.91 ± 0.34 | | 0.5686 ± 0.0061 | 1.5067 ± 0.0311 | | 15.89 ± 0.17 | |  |  |  | |
| BTSTN | **0.2691 ± 0.0242** | **0.9691 ± 0.0543** | **7.58 ± 0.68** | | **0.3012 ± 0.0136** | **1.0256 ± 0.0318** | | **8.42 ± 0.38** | |  |  |  | |

Note: **Values** in bold are the best and values underlined are the second best. Statistical methods labeled with * performed only once.

**TABLE S6** Extrapolation performance of BTSTN on the $\sigma^{28}$-TetR datasets across different missing rates (10%-80%) and Gaussian noise levels (standard deviation from 0.01 to 0.05). Metrics (mean ± SD) are reported in the order of MAE/RMSE/MRE.

| Missing Rate | Gaussian noise (0.01) | | |  | Gaussian noise (0.02) | | | |  | Gaussian noise (0.03) | | |
| --- | --- | --- | --- | --- | --- | --- | --- | --- | --- | --- | --- | --- |
|  | MAE | RMSE | MRE (%) | | MAE | RMSE | | MRE (%) | | MAE | RMSE | MRE (%) | |
| 10% | 0.1789 ± 0.0129 | 0.4579 ± 0.0462 | 4.87 ± 0.35 | | 0.2345 ± 0.0093 | 0.5742 ± 0.0502 | | 6.39 ± 0.25 | | 0.3133 ± 0.0199 | 0.6412 ± 0.0717 | 8.53 ± 0.54 | |
| 20% | 0.1768 ± 0.0042 | 0.4519 ± 0.0114 | 5.59 ± 0.13 | | 0.2223 ± 0.0057 | 0.4794 ± 0.0526 | | 7.03 ± 0.18 | | 0.2883 ± 0.0121 | 0.5752 ± 0.0126 | 9.11 ± 0.38 | |
| 30% | 0.1907 ± 0.0091 | 0.4376 ± 0.0290 | 5.48 ± 0.26 | | 0.2377 ± 0.0017 | 0.4521 ± 0.0288 | | 6.83 ± 0.05 | | 0.3335 ± 0.0233 | 0.5990 ± 0.0292 | 9.59 ± 0.67 | |
| 40% | 0.2037 ± 0.0073 | 0.4731 ± 0.0156 | 5.86 ± 0.21 | | 0.2749 ± 0.0219 | 0.5866 ± 0.0230 | | 7.91 ± 0.63 | | 0.3510 ± 0.0050 | 0.6419 ± 0.0259 | 10.10 ± 0.14 | |
| 50% | 0.2000 ± 0.0228 | 0.4369 ± 0.0858 | 5.80 ± 0.66 | | 0.2654 ± 0.0124 | 0.5224 ± 0.0298 | | 7.70 ± 0.36 | | 0.3879 ± 0.0201 | 0.7229 ± 0.0246 | 11.25 ± 0.58 | |
| 60% | 0.2586 ± 0.0146 | 0.6677 ± 0.0547 | 7.46 ± 0.42 | | 0.3159 ± 0.0074 | 0.7286 ± 0.0326 | | 9.11 ± 0.21 | | 0.4538 ± 0.0111 | 1.0045 ± 0.1051 | 13.08 ± 0.32 | |
| 70% | 0.3145 ± 0.0158 | 0.9886 ± 0.0805 | 8.86 ± 0.45 | | 0.4098 ± 0.0106 | 1.1146 ± 0.0807 | | 11.55 ± 0.30 | | 0.5403 ± 0.0060 | 1.2684 ± 0.0336 | 15.22 ± 0.17 | |
| 80% | 0.4027 ± 0.0174 | 1.1713 ± 0.0472 | 11.26 ± 0.49 | | 0.5358 ± 0.0087 | 1.3098 ± 0.0803 | | 14.98 ± 0.24 | | 0.6207 ± 0.0305 | 1.3708 ± 0.1049 | 17.35 ± 0.85 | |
| Missing Rate | Gaussian noise (0.04) | | |  | Gaussian noise (0.05) | |  | | |  | | | |
|  | MAE | RMSE | MRE (%) | | MAE | RMSE | | MRE (%) | |  |  |  | |
| 10% | 0.4017 ± 0.0302 | 0.7101 ± 0.0679 | 10.94 ± 0.82 | | 0.4750 ± 0.0088 | 0.8130 ± 0.0183 | | 12.94 ± 0.24 | |  |  |  | |
| 20% | 0.4011 ± 0.0099 | 0.7544 ± 0.0267 | 12.68 ± 0.31 | | 0.4682 ± 0.0113 | 0.8322 ± 0.0789 | | 14.79 ± 0.36 | |  |  |  | |
| 30% | 0.4310 ± 0.0169 | 0.7779 ± 0.0196 | 12.39 ± 0.49 | | 0.5592 ± 0.0183 | 0.9390 ± 0.0330 | | 16.08 ± 0.53 | |  |  |  | |
| 40% | 0.4641 ± 0.0355 | 0.8373 ± 0.0749 | 13.35 ± 1.02 | | 0.5598 ± 0.0175 | 0.9839 ± 0.0434 | | 16.10 ± 0.50 | |  |  |  | |
| 50% | 0.4740 ± 0.0220 | 0.8368 ± 0.0329 | 13.75 ± 0.64 | | 0.5709 ± 0.0567 | 0.9767 ± 0.0889 | | 16.56 ± 1.65 | |  |  |  | |
| 60% | 0.6046 ± 0.0529 | 1.1706 ± 0.1090 | 17.43 ± 1.52 | | 0.6363 ± 0.0079 | 1.1295 ± 0.0130 | | 18.35 ± 0.23 | |  |  |  | |
| 70% | 0.6294 ± 0.0216 | 1.3432 ± 0.1180 | 17.74 ± 0.61 | | 0.7335 ± 0.0039 | 1.4948 ± 0.0502 | | 20.67 ± 0.11 | |  |  |  | |
| 80% | 0.7294 ± 0.0206 | 1.5467 ± 0.0317 | 20.39 ± 0.58 | | 0.8572 ± 0.0225 | 1.7087 ± 0.0049 | | 23.96 ± 0.63 | |  |  |  | |

**TABLE S7** Extrapolation performance of SAITS on the $\sigma^{28}$-TetR datasets across different missing rates (10%-80%) and Gaussian noise levels (standard deviation from 0.01 to 0.05). Metrics (mean ± SD) are reported in the order of MAE/RMSE/MRE.

| Missing Rate | Gaussian noise (0.01) | | |  | Gaussian noise (0.02) | | |  | Gaussian noise (0.03) | | |
| --- | --- | --- | --- | --- | --- | --- | --- | --- | --- | --- | --- |
|  | MAE | RMSE | MRE (%) | | MAE | RMSE | MRE (%) | | MAE | RMSE | MRE (%) | |
| 10% | 0.4417 ± 0.0367 | 0.8743 ± 0.0698 | 12.03 ± 1.00 | | 0.4449 ± 0.0232 | 0.8521 ± 0.0611 | 12.12 ± 0.63 | | 0.4633 ± 0.0674 | 0.8837 ± 0.1492 | 12.62 ± 1.84 | |
| 20% | 0.4154 ± 0.0293 | 0.8565 ± 0.0635 | 13.13 ± 0.93 | | 0.4006 ± 0.0160 | 0.8762 ± 0.0094 | 12.66 ± 0.51 | | 0.4508 ± 0.0349 | 0.9436 ± 0.1043 | 14.25 ± 1.10 | |
| 30% | 0.4331 ± 0.0259 | 0.8483 ± 0.0827 | 12.45 ± 0.74 | | 0.4489 ± 0.0330 | 0.9336 ± 0.0361 | 12.91 ± 0.95 | | 0.5551 ± 0.0322 | 1.1166 ± 0.0953 | 15.96 ± 0.93 | |
| 40% | 0.4711 ± 0.0188 | 1.0866 ± 0.0110 | 13.55 ± 0.54 | | 0.4824 ± 0.0163 | 1.1694 ± 0.0596 | 13.88 ± 0.47 | | 0.4810 ± 0.0220 | 1.0591 ± 0.0601 | 13.84 ± 0.63 | |
| 50% | 0.4920 ± 0.0115 | 1.1546 ± 0.0222 | 14.27 ± 0.33 | | 0.4867 ± 0.0081 | 1.0855 ± 0.0431 | 14.12 ± 0.24 | | 0.5187 ± 0.0277 | 1.1348 ± 0.0361 | 15.04 ± 0.80 | |
| 60% | 0.4914 ± 0.0097 | 1.1478 ± 0.0756 | 14.17 ± 0.28 | | 0.5091 ± 0.0373 | 1.1179 ± 0.0542 | 14.68 ± 1.07 | | 0.4887 ± 0.0148 | 1.0503 ± 0.0338 | 14.09 ± 0.43 | |
| 70% | 0.5520 ± 0.0310 | 1.2600 ± 0.0321 | 15.55 ± 0.87 | | 0.5878 ± 0.0295 | 1.3697 ± 0.0763 | 16.57 ± 0.83 | | 0.6162 ± 0.0322 | 1.3698 ± 0.0466 | 17.37 ± 0.91 | |
| 80% | 0.7962 ± 0.2345 | 1.8357 ± 0.2109 | 22.26 ± 6.55 | | 0.6267 ± 0.0161 | 1.6174 ± 0.1188 | 17.52 ± 0.45 | | 0.6221 ± 0.0199 | 1.6413 ± 0.0174 | 17.39 ± 0.56 | |
| Missing Rate | Gaussian noise (0.04) | | |  | Gaussian noise (0.05) | | | |  | | | |
|  | MAE | RMSE | MRE (%) | | MAE | RMSE | MRE (%) | |  |  |  | |
| 10% | 0.4680 ± 0.0399 | 0.8395 ± 0.0519 | 12.75 ± 1.09 | | 0.5294 ± 0.0134 | 1.0516 ± 0.0248 | 14.42 ± 0.36 | |  |  |  | |
| 20% | 0.4553 ± 0.0231 | 0.9301 ± 0.0275 | 14.39 ± 0.73 | | 0.4842 ± 0.0623 | 1.0097 ± 0.1481 | 15.30 ± 1.97 | |  |  |  | |
| 30% | 0.5661 ± 0.0426 | 1.0805 ± 0.0675 | 16.27 ± 1.23 | | 0.5358 ± 0.0367 | 1.0119 ± 0.0731 | 15.40 ± 1.06 | |  |  |  | |
| 40% | 0.5344 ± 0.0312 | 1.1502 ± 0.0531 | 15.37 ± 0.90 | | 0.5394 ± 0.0050 | 1.1993 ± 0.0208 | 15.52 ± 0.14 | |  |  |  | |
| 50% | 0.5126 ± 0.0200 | 1.1616 ± 0.0797 | 14.87 ± 0.58 | | 0.5342 ± 0.0126 | 1.1143 ± 0.0125 | 15.50 ± 0.37 | |  |  |  | |
| 60% | 0.5313 ± 0.0235 | 1.1211 ± 0.0268 | 15.32 ± 0.68 | | 0.6015 ± 0.0362 | 1.1852 ± 0.0417 | 17.34 ± 1.04 | |  |  |  | |
| 70% | 0.6113 ± 0.0076 | 1.3847 ± 0.0525 | 17.23 ± 0.21 | | 0.6729 ± 0.0163 | 1.3995 ± 0.0502 | 18.96 ± 0.46 | |  |  |  | |
| 80% | 0.7118 ± 0.0120 | 1.7922 ± 0.0886 | 19.90 ± 0.33 | | 0.7924 ± 0.0391 | 1.9560 ± 0.1444 | 22.15 ± 1.09 | |  |  |  | |

**TABLE S8** Extrapolation performance of BTSTN on the $\sigma^{28}$-TetR datasets across different missing rates (10%-80%) and time intervals (5, 10, 15, 20). Metrics (mean ± SD) are reported in the order of MAE/RMSE/MRE.

| Missing Rate | Time interval (5) | | |  | Time interval (10) | | |
| --- | --- | --- | --- | --- | --- | --- | --- |
|  | MAE | RMSE | MRE | | MAE | RMSE | MRE |
| 10% | 0.1463 ± 0.0028 | 0.3199 ± 0.0472 | 3.99% ± 0.08% | | 0.1758 ± 0.0100 | 0.4849 ± 0.0644 | 4.79% ± 0.27% |
| 20% | 0.1559 ± 0.0114 | 0.3291 ± 0.0484 | 4.93% ± 0.36% | | 0.1639 ± 0.0156 | 0.4163 ± 0.0534 | 5.18% ± 0.49% |
| 30% | 0.1622 ± 0.0125 | 0.3523 ± 0.0155 | 4.66% ± 0.36% | | 0.1784 ± 0.0057 | 0.4179 ± 0.0146 | 5.13% ± 0.16% |
| 40% | 0.1821 ± 0.0122 | 0.4162 ± 0.0432 | 5.24% ± 0.35% | | 0.2028 ± 0.0126 | 0.4705 ± 0.0657 | 5.83% ± 0.36% |
| 50% | 0.2114 ± 0.0054 | 0.5017 ± 0.0084 | 6.13% ± 0.16% | | 0.2049 ± 0.0067 | 0.4429 ± 0.0391 | 5.94% ± 0.19% |
| 60% | 0.2548 ± 0.0119 | 0.6140 ± 0.0699 | 7.35% ± 0.34% | | 0.2252 ± 0.0204 | 0.5720 ± 0.1362 | 6.49% ± 0.59% |
| 70% | 0.3444 ± 0.0100 | 0.9976 ± 0.0857 | 9.70% ± 0.28% | | 0.3169 ± 0.0153 | 1.0536 ± 0.0696 | 8.93% ± 0.43% |
| 80% | 0.4109 ± 0.0169 | 1.1149 ± 0.0587 | 11.48% ± 0.47% | | 0.3948 ± 0.0118 | 1.1417 ± 0.0446 | 11.04% ± 0.33% |
| Missing Rate | Time interval (15) | | |  | Time interval (20) | | |
|  | MAE | RMSE | MRE | | MAE | RMSE | MRE |
| 10% | 0.1968 ± 0.0047 | 0.5265 ± 0.0490 | 5.36% ± 0.13% | | 0.2578 ± 0.0092 | 0.5749 ± 0.0457 | 7.02% ± 0.25% |
| 20% | 0.1746 ± 0.0074 | 0.4576 ± 0.0399 | 5.52% ± 0.23% | | 0.1974 ± 0.0072 | 0.4617 ± 0.0646 | 6.24% ± 0.23% |
| 30% | 0.1933 ± 0.0046 | 0.4560 ± 0.0104 | 5.37% ± 0.13% | | 0.2285 ± 0.0066 | 0.5482 ± 0.0317 | 6.57% ± 0.19% |
| 40% | 0.2081 ± 0.0082 | 0.4775 ± 0.0124 | 5.99% ± 0.23% | | 0.2320 ± 0.0085 | 0.5674 ± 0.0167 | 6.67% ± 0.25% |
| 50% | 0.2121 ± 0.0035 | 0.5440 ± 0.0347 | 6.15% ± 0.10% | | 0.2393 ± 0.0041 | 0.6091 ± 0.0169 | 6.94% ± 0.12% |
| 60% | 0.2608 ± 0.0137 | 0.7617 ± 0.1209 | 7.52% ± 0.39% | | 0.2861 ± 0.0263 | 0.7096 ± 0.0479 | 8.25% ± 0.76% |
| 70% | 0.3012 ± 0.0133 | 0.9878 ± 0.0253 | 8.49% ± 0.37% | | 0.3375 ± 0.0018 | 1.1742 ± 0.0498 | 9.51% ± 0.05% |
| 80% | 0.3763 ± 0.0143 | 1.1311 ± 0.0491 | 10.52% ± 0.40% | | 0.3996 ± 0.0121 | 1.2446 ± 0.0603 | 11.17% ± 0.34% |

**TABLE S9** Missing rates of annotated metabolites in untargeted metabolomics collected from online mass spectrometry.

| Metabolites | Rate (%) | Metabolites | Rate (%) | Metabolites | Rate (%) |
| --- | --- | --- | --- | --- | --- |
| Pyruvic acid | 8.3 | (R)-2,3-Dihydroxy-3-methylpentanoate | 0.0 | 3-Phospho-D-erythronate | 35.5 |
| DL-Lactic acid | 8.3 | Xanthine | 31.7 | Pantothenic acid | 13.3 |
| 5-Hydroxy-4-pentenoic acid d-lactone | 28.4 | 2,3-Dihydroxybenzoate | 0.3 | Aspartyl-Threonine | 16.6 |
| Succinic anhydride | 2.4 | 2-Oxoadipate | 34.3 | D-glucose 6-P | 23.1 |
| Acetolactate | 25.4 | Alanyl-alanine | 49.7 | Inosine | 17.2 |
| (S)-3-Hydroxybutyric acid | 1.5 | Aminoadipic acid | 21.3 | Adenosine | 81.1 |
| Glyceric acid | 51.5 | 2-(1-Ethoxyethoxy)propanoic acid | 16.9 | Pantetheine | 20.1 |
| gamma-Caprolactone | 34.9 | Phenylpyruvic acid | 5.6 | Endothion | 56.8 |
| L-Proline | 66.3 | 6-Deoxy-L-galactose | 34.6 | Orotidine | 28.4 |
| alpha-Ketoisovaleric acid | 3.8 | D-Phenyllactic acid | 15.7 | Sedoheptulose 7-P | 30.5 |
| L-Valine | 31.4 | N-Acetylleucine | 46.7 | Pyrrolidine | 30.5 |
| Succinic acid | 5.9 | N-Acetylornithine | 37.3 | Dimethylethanolamine | 64.2 |
| 3-Hydroxy-2-methyl-[S-(R,R)]-butanoic acid | 0.6 | 2-Isopropylmalate | 12.7 | 2,5-Dimethyl-1H-pyrrole | 39.9 |
| 3-Mercaptolactic acid | 26.0 | D-Glucono-1,5-lactone | 17.2 | 3-Hexanone | 0.0 |
| Thymine | 36.7 | 4-Hydroxyphenylpyruvic acid | 43.5 | 5-Aminopentanal | 19.2 |
| Dichloroacetate | 5.0 | DL-p-Hydroxyphenyllactic acid | 15.1 | Allopurinol | 35.8 |
| 4-Methyl-2-oxopentanoate | 5.0 | Mannitol | 16.3 | 4-Hydroxynonenal | 0.0 |
| Glutaric acid | 0.3 | 8-Amino-7-oxononanoate | 27.5 | 1-Hexylglycerol | 0.3 |
| (R)-2,3-Dihydroxy-isovalerate | 0.0 | 3-Hydroxysuberic acid | 21.3 | D-Glucose | 14.8 |
| 5-Aceto valeric acid | 29.6 | succinoylpyridine | 43.8 | D-Sorbitol | 35.2 |
| (S)-2-Aceto-2-hydroxybutanoate | 3.6 | 3-Methoxy-4,5-methylenedioxycinnamaldehyde | 32.2 | alpha-D-Ribose 1-methylphosphonate 5-P | 10.1 |
| NMDA | 68.3 | Dihydrolipoate | 56.2 |  |  |
| Citramalic acid | 80.2 | Deoxyribose 5-P | 29.9 |  |  |

**TABLE S10** Comparison of interpolation performance between BTSTN and SAITS on the succinate fermentation dataset across different missing rates from 10% to 80%. Metrics (mean ± SD) are reported in the order of MAE/RMSE/MRE.

| Missing Rate | BTSTN | | |  | SAITS | | |
| --- | --- | --- | --- | --- | --- | --- | --- |
|  | MAE | RMSE | MRE (%) | MAE | | RMSE | MRE (%) |
| 10% | 23812.5326 ± 349.1436 | 91928.7723 ± 686.4297 | 4.73 ± 0.07 | 61411.9746 ± 292.0721 | | 225164.5495 ± 3958.5900 | 12.19 ± 0.06 |
| 20% | 23027.1353 ± 44.1992 | 86601.5004 ± 695.0629 | 4.97 ± 0.01 | 59116.5141 ± 5316.4023 | | 210605.3456 ± 21509.8514 | 12.77 ± 1.15 |
| 30% | 25325.3337 ± 306.0420 | 88902.2327 ± 726.1645 | 5.16 ± 0.06 | 78826.4961 ± 3722.8710 | | 283805.1335 ± 16722.2464 | 16.08 ± 0.76 |
| 40% | 26272.9290 ± 213.3512 | 92584.8873 ± 344.7549 | 5.33 ± 0.04 | 90543.3977 ± 1136.2265 | | 317503.9130 ± 8016.5369 | 18.38 ± 0.23 |
| 50% | 27733.7532 ± 36.5339 | 96914.5184 ± 1098.8727 | 5.53 ± 0.01 | 97167.7409 ± 11535.9367 | | 335050.5146 ± 27190.2115 | 19.39 ± 2.30 |
| 60% | 28610.6006 ± 138.9602 | 100460.1268 ± 582.3167 | 5.76 ± 0.03 | 113415.5015 ± 8270.0549 | | 406004.3492 ± 21466.6244 | 22.84 ± 1.67 |
| 70% | 31240.4898 ± 110.8501 | 113503.6150 ± 1408.8550 | 6.17 ± 0.02 | 162993.7699 ± 19910.4730 | | 588153.4867 ± 74609.3828 | 32.22 ± 3.94 |
| 80% | 34063.2179 ± 190.1275 | 123130.5548 ± 919.5971 | 6.74 ± 0.04 | 245807.2034 ± 2564.5264 | | 871312.2623 ± 14458.0797 | 48.62 ± 0.51 |

**Table S11** Training time of BTSTN for multiple datasets. The average values of stopping epochs and training time are reported in the format of mean ± SD.

| Datasets | Batches | Time points | Variables | Stopping epoch | Training time (s) | Average (s/epoch) |
| --- | --- | --- | --- | --- | --- | --- |
| p53-Mdm2: Sustained | 1 | 1000 | 2 | 64.3 ± 1.2 | 87.4 ± 2.5 | 1.36 |
| p53-Mdm2: Damped | 1 | 1000 | 2 | 63.3 ± 1.2 | 84.9 ± 1.9 | 1.34 |
| $\sigma^{28}$-TetR | 8 | 100 | 4 | 260.0 ± 9.4 | 121.4 ± 4.6 | 0.47 |
| Succinate fermentation | 1 | 338 | 67 | 184.0 ± 10.2 | 390.1 ± 22.7 | 2.12 |
